# DNA Methylation as a Therapeutic Target for Bladder Cancer

**DOI:** 10.3390/cells9081850

**Published:** 2020-08-07

**Authors:** Sandra P. Nunes, Rui Henrique, Carmen Jerónimo, Jesús M. Paramio

**Affiliations:** 1 Cancer Biology & Epigenetics Group-Research Center, Portuguese Oncology Institute of Porto (CI-IPOP), 4200-072 Porto, Portugal; sandra22nunes@gmail.com (S.P.N.); rmhenrique@icbas.up.pt (R.H.); 2 Molecular Oncology Unit, Centro de Investigaciones Energéticas, Medioambientales y Tecnológicas (CIEMAT), 28040 Madrid, Spain; 3 Biomedical Research Institute I+12, University Hospital “12 de Octubre”, 28041 Madrid, Spain; 4 Department of Pathology, Portuguese Oncology Institute of Porto, 4200-072 Porto, Portugal; 5 Department of Pathology and Molecular Immunology, Institute of Biomedical Sciences Abel Salazar—University of Porto (ICBAS-UP), 4050-313 Porto, Portugal; 6 Centro de Investigación Biomédica en Red de Cáncer (CIBERONC), 28029 Madrid, Spain

**Keywords:** bladder cancer, DNA methylation, DNA methyltransferases, nucleoside analogues, therapy

## Abstract

Bladder cancer (BC) is the tenth most frequent cancer worldwide and is associated with high mortality when diagnosed in its most aggressive form, which is not reverted by the current treatment options. Thus, the development of new therapeutic strategies, either alternative or complementary to the current ones, is of major importance. The disruption of normal epigenetic mechanisms, namely, DNA methylation, is a known early event in cancer development. Consequently, DNA methyltransferase (DNMT) inhibitors constitute a promising therapeutic target for the treatment of BC. Although these inhibitors, mainly nucleoside analogues such as 5-azacytidine (5-aza) and decitabine (DAC), cause re-expression of tumor suppressor genes, inhibition of tumor cell growth, and increased apoptosis in BC experimental models and clinical trials, they also show important drawbacks that prevent their use as a valuable option for the treatment of BC. However, their combination with chemotherapy and/or immune-checkpoint inhibitors could aid in their implementation in the clinical practice. Here, we provide a comprehensive review of the studies exploring the effects of DNA methylation inhibition using DNMTs inhibitors in BC, from in vitro and in vivo studies to clinical trials.

## 1. Introduction

Bladder cancer (BC) is estimated to be the tenth most frequent cancer worldwide, being more common in men, in which it is the sixth most frequent cancer and the ninth cause of death due to cancer [1]. At diagnosis, approximately 75% of patients display a non-muscle-invasive BC (NMIBC), whereas 25% are diagnosed with muscle-invasive BC (MIBC) [2]. These differences have important implications regarding the treatment and clinical outcome [3]. NMIBC comprises Ta, T1, and carcinoma in situ (CIS), which do not reach the bladder muscle layer and show relatively good outcomes [4], being treated by transurethral resection (TUR) [3]. Depending on different clinicopathological features, such as number of implants, size, and patient’s age, NMBICs are classified as high or low risk. High risk tumor management includes a regimen of local instillation, mainly of Bacillus Calmette Guérin (BCG), after TUR to prevent recurrences and progression. BCG instillations promote local acute inflammation responses and represent the first approved immunotherapy for human cancers [5]. Recurrence is extremely frequent in NMIBC (up to 70%), and, in a proportion of cases, tumors progress to MIBC (stage T2 or higher) [4,6]. This represents a clinical problem demanding systematic follow-up of patients, frequently requiring cystoscopy, with a serious impact on the patient’s quality of life and generating important costs to health systems [7]. The management of MIBC often requires radical cystectomy and, depending on the clinicopathological characteristics of the neoplasm, platin-based chemotherapy schemes in neoadjuvant or adjuvant settings can be administered [3]. MIBC recurrence and progression are also frequent, with survival at metastatic stage below 5% [6]. In addition to this poor outcome, a percentage of MIBC patients cannot be treated using standard protocols due to comorbidities or other health problems, regularly related to old age at diagnosis. Currently, patients that are considered to be unfit for such protocols have few therapeutic options. The development of immune checkpoint blockade (ICI) with antibodies inhibiting programmed death 1 (PD-1), programmed death-ligand 1 (PD-L1), or cytotoxic T-lymphocyte antigen 4 (CTLA-4) allowed for a substantial improvement in patient survival with good and sustained responses [8]. However, the percentage of patients showing clinical response is still rather limited [9]. Hence, a full understanding of the biological mechanisms underlying the development of BC would not only provide novel biomarkers for early detection but also as candidate therapeutic targets, improving patient survival. To gain better knowledge of BC molecular features, different international initiatives and multiple groups generated large amounts of data including different genomic analyses [10,11]. Such studies revealed the existence of different molecular subtypes and suggested the possibility for designing appropriate subtype-specific therapeutic approaches [12].

**Figure 1:**
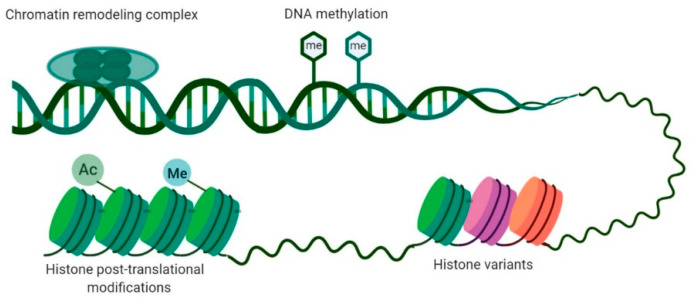
Main epigenetic mechanisms involved in gene expression regulation. DNA methylation consists of the addition of a methyl group in cytosine present in a cytosine–phosphate–guanine (CpG) dinucleotide. Histone post-translational modifications comprise alterations in histone tails such as methylation, acetylation, phosphorylation, and ubiquitination. Histone variants differ in few amino acids from canonical histones and regulate chromatin remodeling and histone post-translational modifications. Chromatin-remodeling complexes regulate the nucleosome structure by removing, relocating, and shifting histones.

One of the most prevalent characteristics of BC is the presence of alterations in the cancer cell epigenetic machinery. The genetic and epigenetic landscapes allow for a cell to acquire an identity associated with a specific tissue, establishing proper organismal physiology [13], and their disruption represents the basis of multiple disorders [13]. Epigenetics comprises the study of heritable alterations that modify gene expression patterns without changing their DNA sequence [13,14,15]. DNA methylation, histone variants, chromatin remodeling, and histone post-translation modifications are the main epigenetic mechanisms with an impact on gene expression (Figure 1) [16]. Although often considered to be independent, different mechanisms cooperate closely in regulating the epigenome [17]. Because the deregulation of such epigenetic mechanisms is implicated in cancer development, in-depth characterization of the cancer epigenome boosted the understanding of tumor initiation and progression [14].

Early in their development and progression, several epigenetic mechanisms are disrupted in BC [18]. For example, mutations in *EP300* and *CREBBP*, two chromatin-remodeling genes, led to the inactivation of the complex’s histone-acetyltransferase domain, resulting in changes in chromatin conformation. Furthermore, a gene expression signature allied with the loss of histone acetyltransferase activity was associated with more aggressive bladder tumors [19]. Interestingly, 89% of MIBC lesions display mutations in the histone-modifying genes [20]. Specifically, a high mutation rate was found in *KMT2D* (also known as *MLL2*) gene and in *KDM6A* (also referred as *UTX*), which encode a histone H3 lysine 4 methyltransferase and a histone lysine demethylase, respectively [21,22,23]. The most explored epigenetic mechanism is DNA methylation [18,24]. By comparing methylation patterns, Wolff et al. found global hypomethylation in NMIBC, whereas hypermethylation patters were more commonly found in invasive tumors, supporting the concept that DNA methylation has an important role in BC development and aggressiveness, constituting a putative target for anti-cancer therapy [24,25].

The main goal of this review is to summarize the impact of aberrant DNA methylation in BC, focusing on therapeutic strategies that target this epigenetic alteration, based on the information provided by preclinical studies, both in vitro and in vivo, and clinical trials.

## 2. DNA Methylation

DNA methylation is the covalent addition of a methyl group at the 5-position carbon of cytosine in a cytosine–phosphatidyl–guanine (CpG) dinucleotide [17]. Methylation occurs mainly at CpG islands—CpG-rich regions (at least 50% of cytosines and guanines) in the genome with a size larger than 200 bp [26,27]. Moreover, methylation can also be found in repetitive sequences such as retrotransposon elements and centromeres, in the X chromosome (leading to its inactivation) and genomic imprinting [28]. Approximately 29,000 CpG islands can be found in the human genome, most commonly in the promoter regions, close to the transcription starting site (TSS) or first exons [27]. Remarkably, 75% of cytosines in CpG dinucleotides dispersed throughout the genome are methylated, whereas cytosines of CpG islands located within gene promoters remain mostly hypomethylated [27,29]. Promoter DNA methylation is classically associated with transcription repression by inducing binding of transcriptional repressors or hampering binding of transcriptional factors [15,17]. In fact, a family of methyl-CpG-binding proteins (MBPs) intervenes in gene silencing by binding to methylated CpGs and recruiting histone modifier enzymes to establish histone post-translation modifications, which further sustain transcriptional repression [30]. DNA methylation can also be found in CpG island shores, 2-kb areas upstream of a CpG island, displaying lower CpG dinucleotide density. CpG island shore methylation is also associated with transcriptional repression [31]. On the other hand, methylation in the gene body was shown to stimulate transcription elongation and to have an impact on splicing, with exons disclosing higher methylation levels than introns [28,32]. Furthermore, tissue-specific methylation seems to be more frequent in intragenic CpG islands [33]. CpG islands in enhancers also influence gene regulation, i.e., hypermethylation is associated with loss of enhancer marks resulting in gene silencing [34].

DNMTs catalyze the covalent bond between the methyl group donated by *S*-adenosyl methionine (SAM) and the unmethylated CpG dinucleotide at the fifth position of the cytosine by positioning the target base into the catalytic pocket of the enzyme using a base-flipping mechanism [35,36,37]. Five DNA methyltransferases (DNMTs) are encoded by the human genome, including DNMT1, DNMT2, DNMT3a, DNMT3b, and DNMT3L [37,38,39]. Specifically, DNMT3a and DNMT3b catalyze de novo DNA methylation, whereas DNMT1 preferentially maintains methylation patterns already existing by copying methylation patterns in the course of replication during embryonic development [37,38,39]. DNMT2 and DNMT3L do not display DNMT catalytic activity, serving as post-transcriptional gene regulation and cofactors of other DNMTs, respectively. By contrast, ten-eleven translocation (TET) enzymes mediate active DNA demethylation through 5-hydroxymethylcytosine (5hmC) [40,41], followed by deamination by activation-induced cytidine deaminase (AID), apolipoprotein B mRNA editing enzyme catalytic subunit 1 (APOBEC1) proteins, and base or nucleotide excision repair [17,42,43].

**Figure 2:**
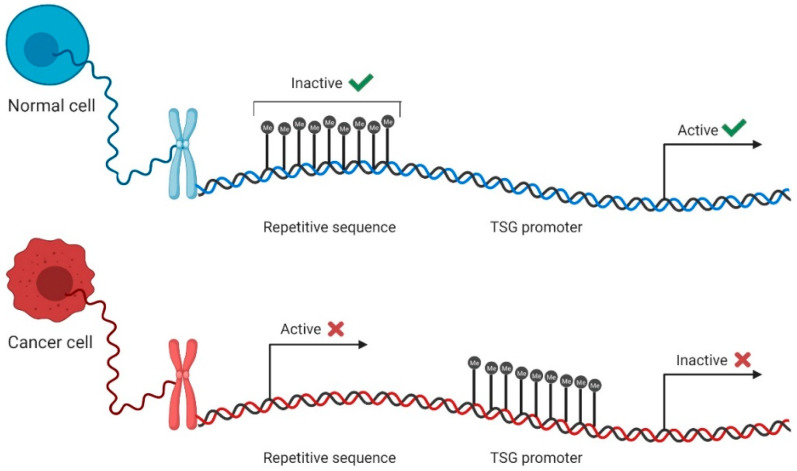
DNA methylation in normal and cancer cells. In normal cells, most repetitive sequences are methylated, whereas the promoters of tumor suppressor genes (TSG) stay unmethylated, remaining active and leading to gene expression (green check mark). Contrarily, in cancer cells, repetitive sequences become unmethylated and active, contributing to genomic instability, and TSG promoters become methylated, inactivating these genes and promoting cell aggressiveness and escape (red cross mark).

Overall, the cancer epigenome is characterized by global hypomethylation, which contributes to the overexpression of proto-oncogenes, an increased mutation rate, and the loss of imprinting [44]. Indeed, a decrease from 80% to 40–60% in the methylation levels from normal to cancer cells was observed [44,45]. More specifically, genomic repetitive regions become unmethylated, stimulating early cancer events through chromosomal instability that result in increased mutation rates, chromosomal rearrangements, and centromere instability (Figure 2) [46,47]. Simultaneously, the promoter hypermethylation of tumor suppressor genes (TSG) is also a frequent event in cancer cells [48], thus contributing to processes such as invasion, metastasis, and angiogenesis [44]. Typically, 5–10% of CpG islands at gene promoters are methylated in cancer (Figure 2) [44].

DNA methylation is a known contributor to BC development [49]. Hence, aberrant DNA methylation was proposed as a promising biomarker for BC detection, prognosis, and therapeutic target [50,51]. For example, a panel comprising *HOXA9*, *PCDH17*, *POU4F2*, and *ONECUT2* methylation detected BC with 90.5% sensitivity and 73.2% specificity in urine samples from Chinese patients presenting hematuria, leading the authors to estimate that about 60% of cystoscopies could be avoided [52]. Furthermore, a methylation panel composed of *GDF15*, *TMEFF2*, and *VIM* discriminated BC patients from healthy controls and prostate or renal cancer patients with a sensitivity of 94% and specificity of 90% in urine sediment samples [53], whereas the UroMark assay based on 150 CpG loci detected BC in voided urine samples with 98% sensitivity and 97% specificity [54]. On the other hand, in an analysis of formalin-fixed paraffin-embedded primary BC tissue samples, methylation of *SFRP5* was associated with recurrence and *CACNA1G* was associated with BC progression [55]. Moreover, DNMTs were shown to be overexpressed in BC, representing an attractive target for anti-cancer therapies [56].

## 3. DNMT Inhibitors

As previously stated, DNA methylation is a reversible alteration that contributes to BC development and progression, and DNMTs are overexpressed in this tumor type [49,56]. Thus, DNMTs constitute attractive targets for cancer treatment, and several “epidrugs” are already approved for the treatment of specific conditions [57]. Indeed, 5-azacytidine (or azacytidine (5-aza, Vidaza^®^)) and 5-aza-2′-deoxycytidine (or decitabine (DAC, Dacogen^®^)) are two DNMT inhibitors approved by the European Medicines Agency (EMA) and Food and Drug Administration (FDA) for the treatment of acute myeloid leukemia (AML), myelodysplastic syndrome, and chronic myelomonocytic leukemia (CMML) [30,57,58]. DNMT inhibitors can be classified, depending on their mechanism of action, as nucleoside and non-nucleoside analogues [24].

### 3.1. Nucleoside Analogues

**Figure 3:**
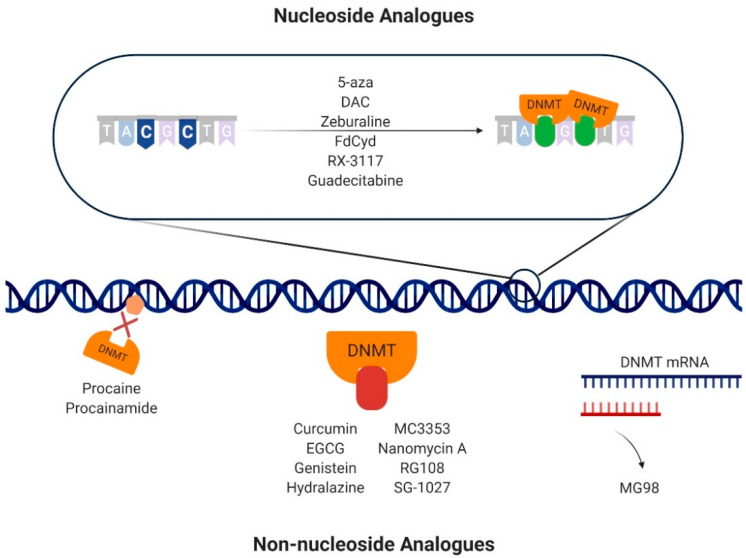
Mechanisms of action of DNA methyltransferase (DNMT) inhibitors. The nucleoside analogues 5-azacitydine (5-aza), decitabine (DAC), zebularine, 5′-fluoro-2′-deoxycytidine (FdCyd), RX-3117, and guadecitabine are integrated into DNA instead of cytosine. When DNMTs bind, a covalent bond is formed between the DNMT and the cytosine analogue. The non-nucleoside analogues have different mechanism of action to achieve inhibition of DNA methylation. Specifically, procaine and procainamide bind directly to the DNA, impeding DNMT binding. Curcumin, epigallocatechin-3-gallate (EGCG), genistein, hydralazine, MC3353, nanomycin A, RG108, and SG-1027 bind directly to the catalytic pockets of DNMTs, hampering their action. MG98 is an oligodeoxynucleotide that binds to the DNMT1 messenger RNA (mRNA) by base pair complementarity, impeding its translation.

Nucleoside analogues (or cytidine analogues) are a group of compounds that integrate into DNA instead of cytosine, resulting in the formation of a covalent bond with a DNMT in the carbon-6 position of the cytosine during the synthesis (S) phase of the cell cycle [57,59] (Figure 3; Figure S1, Supplementary Materials). 5-Aza and DAC are the nucleoside analogues most commonly used as therapeutic agents in cancer [60], with DAC having 90% more demethylating power than 5-aza [61]. Although their mode of action remains controversial, several mechanisms were proposed [59,62,63,64]. After the cellular uptake, mediated by nucleoside transporters, nucleoside analogues are activated through conversion to 5-aza-2′-deoxycytidine-5′-triphosphate, a substrate for the DNA replication machinery, to be incorporated in DNA, replacing cytosine [59]. At that point, DNMTs recognize azacytosine–guanine dinucleotides and catalyze the methylation reaction by forming a covalent bond with the cytosine ring [65,66]. Since azacytosine has a nitrogen substituting the carbon at position 5, the covalent bond cannot be broken, resulting in DNMT inactivation [59]. In addition to DNMT degradation, the complex formed between DNA and DNMT prompts DNA damage by causing double-strand breaks, leading to loss of methylation marks [59,67]. DAC and 5-aza seem to cause different effects in cancer cells. Although they both lead to a depletion of DNMT1 and a decrease in DNA methylation levels in non-small-cell lung cancer cells, 5-aza induces DNA damage, apoptosis, and cell arrest at phase sub-gap 1 (G1), whereas DAC increases the number of cells arrested in gap 2 (G2)/mitosis (M). As a consequence, their effects on gene expression are also different, with 5-aza decreasing the expression of genes related to cell cycle and metabolic processes, while DAC upregulates genes related to cell differentiation [63]. Furthermore, response rates to DAC treatment in AML patients with p53 mutations were higher in comparison with patients with wild-type p53, showing that mutations in p53 may play a role in epigenetically mediated cell death after DAC treatment [62,68]. Remarkably, phosphorylation of DNMT1 by protein kinase δ leads to its faster degradation after treatment with 5-aza or DAC, constituting another possible mechanism via which hypomethylation is achieved [64]. However, 5-aza and DAC are characterized by poor bioavailability, since they are easily degraded by hydrolysis in aqueous acidic or basic environments and have a limited half-life [59,69]. Indeed, both compounds can be deaminated by cytidine deaminase and converted into 5-azauridine, which results in their inactivation [70]. Furthermore, DAC is only incorporated into DNA, whereas 5-aza is incorporated into both DNA and RNA [70] and, since none of the compounds target specific DNMT isoforms, DAC and 5-aza cause significant side effects [70]. Thus, several alternative nucleoside analogues were developed to overcome these limitations.

Zebularine is a cytidine analogue that lacks the amino group at position 4 of the pyrimidine ring. It displays high stability as it inhibits both DNMTs and cytidine deaminase, and it is stable in acidic and neutral pH, enabling oral administration [71]. Moreover, zebularine has low cytotoxicity, which might translate into longer treatments with low doses to maintain a demethylated state [71], as well as an apparent specificity for cancer cells and not fibroblasts [72,73]. Zebularine leads to S-phase delay and cell death in mesothelioma cells [72], causes formation of replication-dependent double-strand DNA breaks [74], and enhances colon cancer cell immunogenicity [75]. Nevertheless, high concentrations of zebularine are needed to achieve demethylation levels similar to those of DAC since it forms a reversible complex with DNMTs, with slow dissociation kinetics [76], hindering the transition to clinical practice [57,71]. 5′-Fluoro-2′-deoxycytidine (FdCyd) is a fluoropyrimidine nucleoside analogue with a mechanism of action similar to 5-aza and DAC [24]. However, it is more stable and induces less toxicity compared to 5-aza and DAC [77]. As these nucleoside analogues are rapidly metabolized, combining them with tetrahydrouridine (THU), a cytidine deaminase inhibitor, was proposed to overcome such limitation [78,79,80,81]. Indeed, co-administration of FdCyd and THU induced plasma concentrations of FdCyd in patients similar to those established as needed for inhibiting DNA methylation in vitro [80].

SGI-110 or guadecitabine is a second-generation hypomethylating agent containing DAC coupled with a deoxyguanosine—a CpG dinucleotide analogue [70]. It is not a substrate for cytidine deaminase, a fact that increases the compound’s exposure time without being inactivated [82]. Guadecitabine was tested in several clinical trials. Specifically, a combination of guadecitabine and irinotecan was shown to be safe and to possess clinical activity in metastatic colorectal cancer patients in a phase I dose escalation study [83]. Likewise, guadecitabine was clinically active in intermediate- and high-risk myelodysplastic syndromes, namely, in patients who did not respond to the currently approved demethylating agents [84]. More recently, an oral formulation of 5-aza, CC-486, was developed and tested in several clinical trials [85,86,87,88]. Some advantages of the oral route include a more convenient and easier administration to monitor, a high exposure time to the drug, and the elimination of local reactions [88]. Although a combination treatment with CC-486 and pembrolizumab was not effective in improving progression-free survival (PFS) in non-small-cell lung cancer patients [86], CC-486 as a single treatment showed clinical activity in nasopharyngeal cancer in a phase I clinical trial [87]. Furthermore, extending doses of CC-486 in low-risk myelodysplastic syndromes patients might provide an effective long-term treatment [88]. RX-3117 (fluorocyclopentenylcytosine) is a novel cytidine analogue with a modified ribose molecule [89]. To be incorporated into DNA or RNA, the RX-3117 molecule is firstly activated through transformation into a triphosphate form by uridine–cytidine kinase 2 [90]. Although RX-3117 is not a substrate for cytidine deaminase [89], other enzymes such as NT5C3 may have a role in breaking an intermediate monophosphate form, resulting in RX-3117 inactivation [90]. RX-3117′s anti-tumor effects were evaluated in nine different xenograft mouse models and showed promise for the treatment of tumors that are sensitive and resistant to gemcitabine [91].

### 3.2. Non-Nucleoside Analogues

Although nucleoside analogues are potent inhibitors of DNA methylation, they lack specificity, resulting in important side effects. To overcome this, non-nucleoside molecules were developed in the last few years. Non-nucleoside analogues comprise all DNMT inhibitors whose mechanism is independent of DNA incorporation [92], including DNA binders, oligonucleotides, natural compounds, SAM competitors, and repurposed drugs, i.e., drugs that were designed for a specific target and treatment but were found to have a novel therapeutic effect [92,93] (Figure 3; Figure S1, Supplementary Materials).

Procaine and procainamide, derivatives of 4-aminobenzoic acid, are FDA-approved drugs as anesthetic and anti-arrhythmic, respectively [94,95]. Both drugs were shown to cause global DNA hypomethylation in MCF7 cells inducing cell growth inhibition [94]. Additionally, procaine reduces the activity of DNMT1 and DNMT3a without influencing their expressions [96]. By directly binding to DNA, procaine and procainamide hamper DNMTs binding, leading to decreased global DNA methylation levels, although to a lesser extent when compared to DAC [94,96]. The antibiotic nanaomycin A also induces global hypomethylation and reactivates TSGs such as Ras association domain family 1 isoform A (RASSF1A) [97]. Remarkably, nanaomycin A showed specificity for DNMT3b inhibition by interacting with key amino-acid residues of this enzyme [98]. Hydralazine is an arterial vasodilator approved for the treatment of severe hypertension and heart failure [99]. It causes re-expression of TSGs by reversing promoter hypermethylation, both in cancer cell lines and in primary tumors [100]. Hydralazine inhibits DNA methylation, although weakly, by interacting with the DNMT active site [99]. Furthermore, hydralazine treatment caused a decrease in cell growth and invasiveness, increased apoptosis, cell-cycle arrest, and lowered DNMT messenger RNA (mRNA) and protein levels [101]. On the other hand, the combined treatment with hydralazine and valproic acid also demonstrated anti-metastatic effects, both in vitro and in vivo [102], and it showed potential clinical benefit in patients with disease progression under chemotherapy [103] including advanced cervical cancer patients [104].

Oligodeoxynucleotides that bind a target mRNA by base pair complementarity also show promise as DNMTs inhibitors [105]. In particular, MG98 is a second-generation 20-nucleotide antisense oligonucleotide that binds specifically to DNMT1 mRNA, causing a decrease in DNMT levels [105]. In an initial phase I clinical trial, MG98 was not well tolerated in high doses, and its anticancer effects were not observed in patients with solid tumors [106]. The lack of clinical activity was also observed in patients with high-risk myelodysplasia and acute myeloid leukemia [107]. However, in another clinical trial in patients with advanced solid tumors using escalating doses, MG98 was well tolerated and showed early clinical benefit [108], similarly to a combination of MG98 and interferon (IFN) α-2β in the treatment of advanced renal cell carcinoma [109]. RG108 is a synthetic molecule considered to be a SAM competitor that targets the binding pockets of DNMTs, establishing covalent bonds and leading to inhibition of their enzymatic activity [110]. Treatment with RG108 caused inhibition of DNMTs and cell growth, and it increased apoptosis in endometrial and prostate cancer cell lines [111,112]. SGI-1027 is a quinolone-based molecule which directly binds to the cofactor-binding site of DNMT3a and to both the cofactor- and the substrate-binding sites of DNMT1 [113]. Treatment of RKO cells with SGI-1027 resulted in demethylation and re-expression of TSGs including p16, mutL homolog 1(MLH1), and metalloproteinase inhibitor 3 (TIMP3), with no significant toxicity [114]. Another quinolone-based molecule, MC3353, caused cell arrest and decreased cell viability in a panel of different cancer cell lines. Furthermore, E-cadherin mRNA and protein levels increased while a reduction of matrix metalloproteinase levels was observed in PC-3 and HCT116 cells, hampering their epithelial-to-mesenchymal transition [115].

Several natural compounds were shown to have DNMT inhibitory activity [24]. The natural polyphenol epigallocatechin-3-gallate (EGCG), found in green tea, was shown to inhibit DNMT activity by interacting with the enzyme catalytic pocket, reactivating the expression of several TSGs by reversing the hypermethylation status [116,117]. EGCG also decreased cell viability, migration and invasion of breast cancer cells, and *SCUBE2* methylation by decreasing DNMT expression and activity [118]. Genistein is an isoflavone present in soybeans which displays anti-cancer activity [24]. In prostate tissue cancer samples, differences in methylation patterns and expressed genes were found comparing patients with genistein supplementation prior to prostatectomy and those receiving placebo [119]. After genistein treatment, DNA methylation levels decreased in several promoters of TSGs such as *ATM*, *APC*, *PTEN*, and *SERPINB5*, with a concomitant increase in the mRNA levels of those genes. Genistein interacts with the catalytic domain of DNMT1, competing with hemi-methylated DNA for the catalytic pocket [120]. Furthermore, in colon cancer cells, genistein demethylated *WIF1* and decreased invasiveness and migration of cancer cells by reducing expression of matrix metalloproteinases 2 and 9 [121]. Curcumin, a polyphenol compound with anti-inflammatory properties, also influences methylation status. In colorectal cancer cells, exposure to curcumin led to demethylation of specific CpG loci without inducing global methylation changes [122].

## 4. DNA Methylation as a Therapeutic Target in BC

### 4.1. Preclinical Studies

A summary of the preclinical studies testing DNMT inhibitors in BC is depicted in Table 1. The effects of 5-aza were evaluated in cell lines in vitro and in a tumor xenograph model. 5-Aza was shown to inhibit cell proliferation and arrest cells at G0/G1, whereas volume and weight of tumor xenografts in mice were reduced. Interestingly, DNMT3a and DNMT3b expressions were also reduced after 5-aza treatment, causing the re-expression of hepaCAM, a TSG [56]. DAC treatment also led to an increase in hepaCAM expression in T24 and BIU87 cells, associated with arrest at G0/G1 phase [123]. In a canine model of invasive urothelial carcinoma, 5-aza disclosed anti-tumor effects, with 22.2% of the dogs demonstrating partial response and 50% depicting stable disease [124].

Non-toxic concentrations of DAC were used to treat four BC cell lines in order to evaluate the impact of hypomethylation in the BC cell transcriptome. Notch receptor 1 (NOTCH1) expression increased after treatment with DAC, in parallel with 50% demethylation of the promoter and enhancer regions. Interestingly, DAC-treated cells displayed morphological changes, i.e., cell enlargement compared to the controls. To explore these effects, the active intracellular domain of NOTCH1, ICN1, was overexpressed in cell lines and a decrease in cell proliferation was observed. Moreover, there was an increase of interleukin (IL)-6, probably due to a rise in ICN1 expression and double-stranded RNA levels [125]. ICN1 overexpression also led to a decrease in basal stem-like cells, as assessed by the decreased cytokeratin 5 (CK5) levels, contributing to a more differentiated cell state [125], which may prevent BC progression [126]. DAC was also shown to inhibit cell proliferation, migration, and invasiveness, inducing apoptosis in T24 cells while increasing the expression of the TSG Maspin [127]. Another gene that appears to be regulated by methylation in BC is *BTG2*. After treating EJ cells with DAC, BTG anti-proliferation factor 2 (BTG2) expression increased mainly due to DNMT1 inhibition, and cell growth decreased. Furthermore, H3K9me2 levels decreased, whereas H3K4me3 increased, leading to an open chromatin state in the promoter and intronic region of *BTG2* [128]. Apoptotic peptidase activating factor 1 (APAF-1) and death associated protein kinase 1 (DAPK-1) expressions also increased after DAC treatment in both RT4 and T24 cells, whereas zebularine caused an increased expression of those genes in cell line T24, which is p53 mutated [129]. Remarkably, Cheng et al. showed that treatment of T24 cells with zebularine for 48 h was not enough to maintain a long-term hypomethylated state and sustained p16 expression, as the percentage of methylation at day 0 was 97% and decreased to 75% at day 3. Inversely, a continuous treatment for 40 days, renewing dosage every three days, repressed cell growth and sustained p16 expression, while DNMT1 recovery was null. However, sequential treatment with DAC and zebularine was the most effective in increasing and retaining p16 expression, maintaining promoter hypomethylation, and hampering re-methylation patterns observed before treatment [130]. Therefore, to increase treatment efficacy with this type of drug, a prolonged treatment seems to be pivotal.

The epigenetic regulation of Wnt inhibitory factor 1 (Wif-1), an antagonist of the Wnt pathway, important for carcinogenesis, was explored in four BC cell lines. DAC treatment led to increased Wif-1 mRNA levels with a simultaneous decrease in promoter methylation levels. Furthermore, Wif-1 expression was primarily regulated by DNA methylation and not genetic alterations [131]. DAC treatment in T24 cells induced the expression of several genes related to the IFN pathway, which could theoretically lead to inhibition of tumor cell growth [132]. Interestingly, Velicescu et al. showed that de novo methylation does not occur in non-dividing BC cells. Treating T24 cells with DAC allows for cell arrest at G0/G1 and determines how much time is necessary for re-methylation to occur. Indeed, no re-methylation was found in CpG islands, whereas various degrees of methylation reappeared in CpG poor regions. Furthermore, DNMT1 and 3b3 protein levels were not detected, whereas DNMT3a mRNA levels were maintained after day 10 of the experiment. This result demonstrates that DNMT3a might catalyze a de novo methylation in CpG poor regions outside the S phase of the cell cycle [133]. In another study, the carcinogen *N*-butyl-*N*-(4-hydroxybutyl)nitrosamine) (BBN) was used to induce bladder tumors in mice. These tumors showed downregulation of several genes including GSTM1, which seems to be regulated, in part, by DNA methylation, since treatment with DAC in 5637 cells increased GSTM1 expression [134]. Kawakami et al. reported for the first time that *MSH3* epigenetic regulation by means of DNA methylation might contribute to gene silencing, being implicated in BC carcinogenesis [135].

Recently, a study comprising a wide range of different cancer cell lines, containing BC, showed that DNMT inhibitors, including DAC and 5-aza, increase methylation levels throughout the cancer epigenome. Specifically, in BC cell lines, DAC treatment increased methylation levels of 616 common CpGs and decreased methylation levels of 590 different CpGs, demonstrating that the DNMT inhibitor mechanism of action is complex and requires further exploration [136].

After treatment with S110, global methylation levels decreased, concomitantly with increased p16 expression [137]. The effects of S110 in vivo were also assessed in a tumor xenograft mouse model using EJ6 cells. Tumor-free animals tolerated S110 better than DAC, with less weight loss and mortality. S110 failed to cause reduction in tumor sizes, yet their growth rate was lower and p16 expression was induced [138]. The development of DAC and 5-aza variants could potentially increase their half-life, improving bioavailability and therapeutic efficacy.

Procainamide and hydralazine inhibitory effects on DNA methylation were studied in T24 cells. Both compounds decreased the methylation levels of TSGs *p16* and *RARβ*, which associated with their re-expression, both at the transcript and at the protein levels. Interestingly, hydralazine treatment maintained p16 reactivation for longer than DAC [100]. A novel strategy to inhibit DNMT1, using an essential enzyme for cancer cell viability [139] highly expressed in BC [140], was proposed using DNAzymes. A DNAzyme is a stable DNA molecule with catalytic activity that targets specific RNA molecules leading to their destruction [141,142]. The DNAzyme DT433, constructed and selected to target DNMT1, displayed effects that were similar to those of 5-aza. Additionally, DT433 led to an increase in p16 expression and inhibition of cell proliferation [142]. Novel strategies applying repurposed drugs or DNAzymes to achieve DNMT inhibition constitute interesting alternatives to the demethylating drugs already approved. On the one hand, DNAzymes showed that increasing specificity toward the target may be achieved, whereas repurposed drugs, with negligible toxicity and known safety profiles, may be administered for longer periods and be considered as the next step for DNA methylation inhibition as anti-cancer therapy.

### 4.2. Combination Studies

The anti-tumor effects of 5-aza, trichostatin A (TSA), and FK228 (the latter is a class I histone deacetylase (HDAC) inhibitor) were evaluated individually and in combination in a set of BC cell lines, as well as xenograft and orthotopic mouse models. The combination 5-aza and FK228 was shown to be toxic for 90% of the BC cells, inducing apoptosis and decreasing the G2/M cell population. The same combination in in vivo models led to a decrease in tumor size, mainly due to the effect of FK228 alone [147]. Combination treatments using 5-aza and TSA also reduced the cell number and a shift in the expression of proteins that regulate the cell cycle in canine BC cell lines [148]. More recently, combination of DAC and entinostat, another HDAC1 inhibitor, was tested in bladder cell lines, including two cisplatin-sensitive (J82 and RT112) and one cisplatin-resistant (J82CisR) cell lines, as well as one urothelial cell line isolate from normal tissue (HBLAK). The combination treatment did not revert cisplatin resistance of J82CisR, although treatment with both drugs promoted cell growth arrest. Additionally, increased apoptosis was observed, related to caspases 3 and 7, prompting cell arrest at G2/M transition. Forkhead box O1 (FoxO1) expression increased after the combination treatment, as well as BIM and p21, along with a decrease in survivin expression [149].

Several strategies using epigenetic drugs were devised to overcome chemoresistance and increase therapy success. Ramachandran et al. showed that pre-treatment with 5-aza followed by cisplatin or docetaxel exposure increased cytotoxicity in BC cells. Moreover, in UMUC3 cells that developed resistance to cisplatin or docetaxel in vitro, pre-treatment with 5-aza resulted in 44% and 55% of cytotoxicity in cisplatin- and docetaxel-resistant cells, respectively, in opposition to 2% with no pre-treatment [150]. Pre-treatment with DAC also enhanced cytotoxicity of cisplatin and doxorubicin in BC cells. Furthermore, RASSF1A expression was observed concomitantly with activation of the Hippo pathway [151]. Interestingly, UMUC14, RT4, 96-1, and 97-1 cell lines displayed low sensitivity to cisplatin, being associated with high *HOXA9* methylation levels. This was also verified in MIBC patients in which high *HOXA9* methylation levels were associated with resistance to chemotherapy. In line with other studies, DAC led to a 4–5-fold decrease in half maximal inhibitory concentration (IC_50_) for cisplatin, vinblastine, doxorubicin, and etoposide in BC cells [152]. Wu et al. demonstrated for the first time that DAC treatment reduced the cancer stem-cell population in mice. Specifically, in a BNN-induced mouse model of BC, DAC alone or in combination with cisplatin or gemcitabine led to a decline in the keratin 14 (KRT14)+ cell population, which originates from the bladder urothelium [153]. Consistently, the percentage of SRY-box transcription factor 2 (SOX2)+ cell population assumed to be responsible for the spread of the tumor [154] was also lower. Curiously, the percentage of such cells increased in mice treated with chemotherapy only [155]. These results were replicated in patient sample-derived xenografts, demonstrating that a combination treatment of DAC with chemotherapeutic agents might constitute a valuable option for BC therapy [155]. Another study showed that a quadruple combination therapy comprising gemcitabine, cisplatin, DAC, and TSA increased apoptosis via cyclin D1 (CCND1) downregulation, DNA fragmentation, and caspase 3 expression in T24 cells. On the other hand, a cell proliferation reduction and lower BCL2L1 mRNA levels were observed after treatment [156].

A novel dual inhibitor, CM-272, targeting G9a, a histone methyltransferase that catalyzes H3K9me2, and DNMTs demonstrated activity against a wide range of cancer cells [157]. Specifically, treatment of hematologic malignancies cell lines with concentrations in the nanomolar range led to a global decrease in H3K9me2 and 5-methylcytosine levels, a reduction in cell proliferation, induction of cell-cycle arrest and apoptosis, and a decrease in TSG promoter methylation. Interestingly, CM-272 also induced an IFN-γ type I response and immunogenic cell death. In an in vivo mouse model, CM-272 was safe to administer, with an increased overall survival of the treated mice [157]. Furthermore, CM-272 was also active against hepatocellular carcinoma cell lines [158]. Recently, the effects of CM-272 were evaluated in in vitro and in vivo models of BC. CM-272 in combination with cisplatin led to inhibition of cell proliferation, which was also verified in a BC xenograft mouse model, leading to a decreased tumor growth, whereas apoptosis and autophagy were increased [159]. These effects were also observed in a quadruple-knockout transgenic mouse model of advanced BC. Remarkably, BC cells treated with CM-272 showed upregulation of genes linked to the immune response, including IFN-α and γ, and tumor necrosis factor (TNF)-α, probably through induction of an endogenous retrovirus response [159]. Taking into account these results, the combination of CM-272 with anti-PD-L1 in the quadruple-knockout mouse model was explored. A sustained response to the combination treatment was observed, with the number of animals developing tumors or metastases being lower in the combination group when compared with the group treated with anti-PD-L1 monotherapy [159]. Remarkably, CM-272 treatment led to an immune reactivation of tumor cells, turning “cold” tumors into “hot” ones [159]. A brief overview of the main studies exploring the combination therapies in BC experimental models can be found in Table 2.

### 4.3. Clinical Studies

A non-randomized multicenter phase II study evaluated the effects of FdCyd in THU [80,162]. The safety, maximum tolerated dose (MTD), pharmacokinetics, and pharmacodynamics of this combination were determined in a previous phase I clinical trial (FyCyd 100 mg/m^2^/day and THU 350 mg/m^2^/day) [81]. Patients with metastatic or unresectable breast cancer (*n* = 29), head and neck cancer (*n* = 21), non-small-cell lung cancer (*n* = 25), and urothelial cell carcinoma (*n* = 18) which endured progression after at least one line of standard therapy were included in this study. The combination was well tolerated, and urothelial carcinoma patients showed some clinical responses, with an objective response rate (ORR) of 5.6%, a progression-free survival (PFS) of 3.6 months, and a four-month PFS probability of 42%. Furthermore, increased p16 expression was observed in cytokeratin-positive circulating tumor cells (CTCs) of some patients, although it did not associate with clinical response (Table 3) [162].

The pharmacokinetics, toxicity, and clinical relevance of combining 5-aza and sodium phenylbutyrate, a first-generation HDAC inhibitor, was assessed in 34 patients with refractory solid tumors with no curative options, including two BC patients. Twenty-seven patients were eligible to proceed with treatment in three different regiments. The treatment was well tolerated with the most common toxicity effects including neutropenia, anemia, nausea, vomiting, transaminase elevation, and edema. The results of the study were mostly disappointing, with only one leiomyosarcoma patient displaying stable disease for 4.5 months and the rest disclosing disease progression. Although inhibition of DNMT activity was observed in two patients, this drug combination failed to show clinical benefit (Table 3) [163].

In a phase Ib clinical trial, the effects of CC-486 to potentiate carboplatin or paclitaxel protein-bound particles (ABI-007) in 169 patients with relapsed or refractory solid tumors including BC were evaluated. Two arms were defined: arm A comprising treatment with CC-486 and carboplatin and arm B including combination treatment of CC-486 and ABI-007 [164]. Preliminary results showed that CC-486 (200 and 300 mg) was well tolerated, with treatment-emergent adverse events (TEAEs) including anemia and neutropenia in about 50% of the patients in arm A and nausea/vomiting and peripheral neuropathy in patients of arm B. Five patients in arm A showed stable disease whereas three patients displayed partial response, with both combinations disclosing clinical value. Interestingly, peripheral blood mononuclear cells (PBMCs) were found to be hypomethylated. A recommended phase II dose (RP2D) study will comprise an expansion of cohorts and the combinations of 300 mg of CC-486 with carboplatin and 200 mg of CC-486 with ABI-007 [165].

Other clinical trials are at this date assessing the clinical benefit of combining immune checkpoint inhibitors with epigenetic drugs. In a recently completed study, the combination of pembrolizumab, epacadostat [indoleamine 2,3-dioxygenase 1 (IDO1)-selective inhibitor], and 5-aza was assessed in a phase I and II trial enrolling 70 patients, including urothelial cancer patients [166]. Furthermore, in a currently recruiting phase II clinical trial, the biological and clinical efficacy of atezolizumab (which targets PD-L1) in combination with guadecitabine will be evaluated in 53 patients with checkpoint inhibitor-refractory or -resistant urothelial carcinoma (Table 3) [167].

## 5. Conclusions

**Figure 4:**
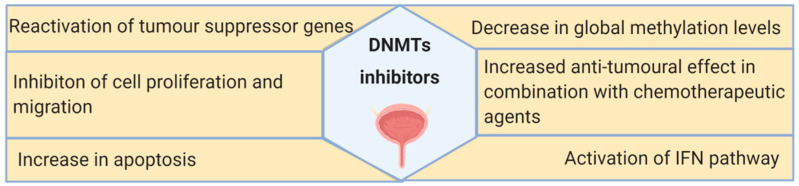
Summary of the main effects of DNMT inhibitors observed in bladder cancer in vitro and in vivo models, as well as in clinical trials.

DNA methylation machinery is a promising target for BC treatment. Among the anti-tumor effects caused by DNMT inhibitors, the most frequently reported are reactivation of TSGs and inhibition of tumor cell growth (Figure 4). However, DNMT inhibitors still present several drawbacks that preclude their use for BC treatment, including the reversal of the inhibitory effects after drug withdrawal, short half-life, and significant toxicity. To overcome these, combination therapies comprising DNMT inhibitors, chemotherapeutic agents, and, more recently, immune checkpoint inhibitors might constitute a valid option for BC patients, as demonstrated by the currently ongoing clinical trials. Furthermore, the discovery of novel biomarkers to select patients more likely to respond to those therapies and the evaluation of the long-term effect of this class of compounds are required for the transition into clinical practice.

In conclusion, modulation of the epigenetic landscape of BC tumors constitutes an encouraging alternative to the currently available therapeutic strategies for BC patients, requiring further exploitation in a clinical trial setting, aiming at a future implementation in clinical practice.

## Figures and Tables

**Table 1:** Summary of pre-clinical studies targeting DNMTs in bladder cancer (BC).

Drug	Model	Concentration	Treatment Scheme	Effects	Year	Reference
DAC	T24	1 µM	1 day	↑ Gene expression related to IFN pathway	2002	[132]
DAC	T24	3 µM	1 day	No remethylation in CpG islands in the absence of cell division	2002	[133]
Hydralazine and procainamide	T24	10 µM	5 days	↓ *p16* and *RARβ* methylation levels ↑ p16 and RARβ expression	2003	[100]
DAC	TCC and UMUC	5 µM	n.a.	↑ MSH3 mRNA levels	2004	[135]
Zebularine	T24	100 µM	Every 3 days for 40 days	↓ Global methylation levels ↑ p16 expression	2004	[130]
DAC	J82C, T24C, TCC, and UMUC	5 µM	3 days	↑ Wif-1 mRNA expression levels	2006	[131]
DAC Zebularine	RT4 and T24	2 µM 100 µM	2 days Every 3 days for 7 days	↑ Cells doubling time ↑ APAF-1 and DAPK-1 expression	2006	[129]
S110	T24	0.1–10 µM	Every 3 days for 6 days	↓ Global methylation levels ↑ p16 expression	2007	[137]
DAC	BIU87	0.1–5 µM	3 days	Re-expression of RASSF1A	2009	[143]
DAC	BOY	1 µM	4 days	↑ COL1A2 expression	2009	[144]
DAC	BOY, T24, and UMUC	10 µM	7 days	↑ FHL1 mRNA expression levels	2010	[145]
S110	Mouse tumor xenograft	10 mg/kg	Daily injection for 6 days	↓ Tumor growth rate ↑ p16 expression	2010	[138]
5-Aza	Dogs with naturally occurring invasive urothelial carcinoma	0.1–0.3 mg/kg	Two doses schedules: Everyday days 1 to 5 or days 1 to 5 and 15 to 19 Each cycle 28 days	22.2% Tumor partial response 50% Stable disease 22.2% Progressive disease	2012	[124]
DAC	BIU87 and T24	0.1–10 µM	3 days	Cell arrest at G0/G1 ↑ hepaCAM expression	2013	[123]
DAC	T24	0.25–2 µM	2 days ^1^	↑ Maspin expression levels ↓ Cell proliferation, migration and invasion	2013	[127]
DAC	5637	1–3 µM	6 days	↑ GSTM1 expression	2014	[134]
DAC	EJ	1 µM	Every day for 3 days	↓ Cell tumorigenesis and invasiveness Cell arrest at G2/M ↑ BTG2 expression	2014	[128]
DNAzyme	T24	n.a.	n.a.	↓ Cell proliferation ↑ p16 expression	2015	[142]
5-Aza	BIU87, EJ, and T24 Mouse tumor xenograft	0.5–7 µM n.a.	1–4 days Every 3 days for 18 days	↓ Cell proliferation ↓ Tumor volume and weight	2016	[56]
DAC	T24 and J82	0.3 µM	1 day	↓ *RSPH9* methylation levels	2016	[146]
DAC	BLCAb001 (B01), BLCAb002 (B02), HT1376, and T24	0.1–1 µM	Every 2 days for 5 days	↑ NOTCH1 expression ↓ CK5 positive cells ↑ IL-6 release	2017	[125]
DAC	T24	1 µM	1 day	↓ Methylation of 590 CpGs ↑ Methylation of 616 CpGs	2019	[136]

^1^ For migration and invasiveness assays. Abbreviations: 5-aza—5-azacytidine; APAF-1—apoptotic peptidase activating factor 1; BTG2-BTG anti-proliferation factor 2; CK5—Cytokeratin 5; CpG—Cytosine–phosphatidyl–guanine; COL1A2—collagen type I alpha 2 chain; DAC—decitabine; DAPK-1—death associated protein kinase 1; FHL1—four and a half LIM domains 1; GSTM1—glutathione S-transferase mu 1; IFN—Interferon; IL-6—Interleukin 6; MSH3—mutS homolog 3; n.a.—not available; NOTCH1—Notch receptor 1; RARβ—retinoic acid receptor beta; RASSF1A—Ras association domain family 1 isoform A; ↑—increase, ↓—decrease.

**Table 2:** Summary of combination studies targeting DNMTs in BC.

Drug	Model	Concentration	Treatment Scheme	Effects	Year	Reference
5-Aza and FK228	253J, T24, TCCSUP, UMUC3, and WH	5-aza 1–25 µM FK228 0.25-5 ng/mL	3 days	↑ Apoptosis ↓ G2/M cell population	2007	[147]
DAC and cisplatin	253J, RT112, T24, and TCCSUP	DAC 0.1–8 µM Cisplatin 0.25–2 µg/mL	3 days	↑ Cell arrest at G2/M ↓ Proliferative ability ↑ Apoptosis ↑ Susceptibility to cisplatin	2008	[160]
5-Aza, cisplatin, and docetaxel	T24, TCCSUP, and UMUC3	5-Aza 0.6 µM Cisplatin 1 µM Docetaxel 5 nM	5-Aza for 72 h followed by cisplatin or docetaxel for 72 h	↑ Cell toxicity with combined treatment	2011	[150]
5-Aza and TSA	K9TCC, K9TCC-PU-Nk, and K9TCC-PU-Sh	5-Aza 1–50 µM TSA 0.5 µM	2 days	↑ p16 expression ↓ Cell number ↓ Cyclin D1, p21, pRb, survivin, and PARP expression	2013	[148]
DAC, TSA, cisplatin, and gemcitabine	T24	Gemcitabine 2.5 µM Cisplatin 1.25 µM DAC 10 µM TSA 300 nM	Gemcitabine and cisplatin for 48 h followed by DAC for 48 h and TSA for 6 h	↑ Apoptosis ↓ Cell proliferation ↑ DNA fragmentation ↑ CASP-3 mRNA levels ↑ GSK3β mRNA levels ↑ Canonical Wnt pathway ↓ BCL2L1 mRNA levels	2014	[156]
DAC, cisplatin, doxorubicin, etoposide, and vinblastine	96-1, 97-1, RT4, and SW1710	DAC 100 nM Cisplatin 0.3–3000 µM Etoposide, vinblastine, and doxorubicin 0.1–1000 µM	DAC for 120 h followed by chemotherapeutic drugs for 48 h	↓ IC_50_ of chemotherapeutic drugs with combined treatment	2016	[152]
DAC, cisplatin, and doxorubicin	HT1376 and T24	DAC 1 and 5 µM Cisplatin and doxorubicin 1–10 µg/mL	DAC for 72 h followed by cisplatin or doxorubicin for 72 h	↑ Cell toxicity ↑ RASSF1A expression Hippo pathway activation	2018	[151]
DAC, cisplatin, and gemcitabine	5637 and SCaBER	DAC 100 nM Cisplatin 100–150 ng/mL Gemcitabine 2–150 ng/mL	Daily for 72 h	↑ Apoptosis ↓ Ratio of CD44v6+ and ALDH+ cells ↑ SOCS3 expression	2019	[155]
	BNN-induced mouse model	Cisplatin 2.5 mg/kg Gemcitabine 120 mg/kg DAC 0.05–0.2 mg/kg	Cisplatin weekly for 3 weeks Gemcitabine weekly for 3 weeks DAC once daily for 5 consecutive days and 3 times per week	↓ Number of invasive tumors ↓ Cancer cells proliferation ↑ Apoptosis ↓ KRT14+ expressing cells ↓ SOX2+ expression cells ↓ STAT3 phosphorylation		
	Patient sample-derived xenografts					
DAC and cisplatin	CR-T24 and T24	DAC 2 µM Cisplatin 1 µg/mL	DAC for 48 h followed by cisplatin for 24 h	↓ Colonies formation ↑ Tap73 expression ↑ Cisplatin response	2019	[161]
CM-272 and pembrolizumab	Quadruple-knockout transgenic mouse model of metastatic BC. Cre-dependent inactivation of *Pten*, *Trp53*, and *Rb1* specifically in urothelial cells (AdK5Cre) of Rbl1-deficient mice	CM-272 5 mg kg^−1^ Anti-PD-L1 200 μg per injection	Treatment of mice started at the time of tumor detection. CM-272 intraperitoneally 5 days per week. Anti-PD-L1 once a week for a total of 3 injections.	Extensive immune infiltrations comprising CD3+, CD8+, and NK cells Low number of animals showing tumor or metastasis evidence. Prolonged anti-tumor effect without any treatment	2019	[159]
DAC and entinostat	J82, J82CisR, HBLAK, and RT112	DAC 0.1 and 1 µM Entinostat IC_50_ according to cell line	DAC for 48 h followed by entinostat for 48 h	Growth inhibition ↑ Apoptosis ↑ Cell arrest in G2/M transition ↑ FoxO1 expression ↑ BIM and p21 expression ↓ Survivin expression	2020	[149]

Abbreviations: 5-aza—5-azacytidine; CASP-3—caspase 3; DAC—decitabine; FoxO1—forkhead box O1; GSK3β—glycogen synthase kinase 3 beta; KRT14—keratin 14; PARP—poly(ADP-ribose) polymerase; RASSF1A—Ras association domain family 1 isoform A; SOCS3—suppressor of cytokine signaling 3; SOX2—SRY-box transcription factor 2; STAT3—signal transducer and activator of transcription 3; Tap73—tumor protein P73; TSA—trichostatin A. ↑—increase, ↓—decrease.

**Table 3:** Clinical trials in BC using DNMT inhibitors.

Drug	Phase (ID)	Status	Enrollment	Schedule	Results	Period	Reference
5-Aza and sodium phenylbutyrate	I (NCT00005639)	Completed	Patients with diagnosis of a refractory solid tumor malignancy with no curative options including BC (*n* = 34)	Regimen A: Low-dose of 5-aza with intermittent phenylbutyrate 400 mg/m^2^/day over 24 h on days 6 and 13. Regiment B: 5-aza 75 mg/m^2^/day for 7 days, followed by two different doses of phenylbutyrate starting on day 8 and continuing for 7 days. Each cycle lasts 35 days for A and B. Regiment C: 2 different daily doses of 5-AC for 21 days and phenylbutyrate 400 mg/m^2^/day over 24 h once per week. Each cycle lasts 42 days.	Three doses were well tolerated. Common toxicities included bone marrow suppression-related neutropenia and anemia. One patient showed stable disease; the remaining did not show any clinical response.	2000–2005	[163]
DAC	I (NCT00030615)	Completed	Advanced metastatic solid tumor patients after other standard therapies fail including BC (*n* = 24)	DAC intravenous (IV) over 30 min on days 1–5 weekly for 4 weeks. Course repeated every 6 weeks in the absence of disease progression or unacceptable toxicity.	Not available	2001–2008	[168]
FdCyd and THU	II (NCT00978250)	Completed	Metastatic or unresectable solid tumors including urothelial transitional cell carcinoma (*n* = 18), whose disease progressed after at least one line of standard therapy.	FdCyd (100 mg/m^2^/day) by 3 h intravenous infusion and THU (350 mg/m^2^/day) 20% as a bolus, with the remaining co-administered with FdCyd over 3-h infusion on days 1–5 and 8–12 of each 28-day cycle.	Co-administration with THU was shown to increase the area under the curve of FdCyd more than 4-fold. Combination was well tolerated. ORR of 5.6%, PFS of 3.6 months, and 42% of 4-month PFS probability for urothelial cancer patients.	2009–2019	[162,169]
CC-486, carboplatin, and paclitaxel protein-bound particles (ABI-007)	I (NCT01478685)	Completed	Patients with relapsed or refractory solid tumors including urinary bladder neoplasms (*n* = 169)	Arm A: CC-486 (doses between 100–300 mg) was administered orally daily either 14 or 21 days. Carboplatin was given by intravenous (IV) infusion once every 21 days Arm B: CC-486 (doses between 100–300 mg) was administered orally daily for either 14 or 21 days ABI-007 was administered by intravenous (IV) infusion on two of every three weeks Arm C: CC-486 (doses between 100–300 mg) was administered orally daily for either 14 or 21 days.	CC-486 dosed 14/21 days was tolerated as a priming agent with carboplatin and ABI-007. Both combinations show evidence of clinical activity.	2011–2015	[164,165]
RX-3317	I (NCT02030067)	Completed	Patients with advanced or metastatic solid tumors including advanced BC (*n* = 124)	A cycle was 4 weeks, with up to 8 cycles. RX-3117 dosing was given 3 times each week for 3 weeks followed by 1 week off treatment. All subjects were followed for at least 30 days after the last dose of RX-3117.	Not available	2013–2019	[170]
CC-486	I (NCT02223052)	Completed	Subjects with hematologic or solid tumor malignancies including BC patients (*n* = 89)	Arm 1: Two 150-mg tablets of CC-486 on day 1 and 1 × 300 mg CC-486 on day 2 Arm 2: 1 × 300 mg tablet of CC-486 on day 1 and 2 × 150 mg CC-486 on day 2.	Not available	2014–2018	[171]
SGI-110, gemcitabine, and cisplatin	Ib/IIa (2015-004062-29)	Recruiting	Urothelial BC patients with stages T2-4aN0M0 (*n* = 20)	Arm 1: SGI-110 days 1–5 at the determined dose, gemcitabine 1000 mg/m^2^ days 8 + 15, cisplatin 70 mg/m^2^ day 8. 3–4 cycles of 21 days each. Arm 2: Gemcitabine 1000 mg/m^2^ days 8 + 15, cisplatin 70 mg/m^2^ day 8 3–4 cycles of 21 days each for both arms. 3–4 cycles of 21 days each.	Not available	2015–present	[172]
75 approved agents	II (NCT02788201)	Completed	Patients with a diagnosis of metastatic, progressive urothelial carcinoma of the bladder, urethra, ureter, or renal pelvis (*n* = 8)	COXEN algorithm was used to determine the best therapy from among 75 FDA-approved agents (single agent or combination). Patients had regular visits for blood, urine, and tumor scans.	Not available	2017–2019	[173]
Azacitidine, pembrolizumab, and epacadostat	I (NCT02959437)	Completed	Subjects with advanced or metastatic solid tumors including BC patients (*n* = 70)	Five doses of azacitidine were administered by subcutaneous injection or intravenously (IV) over days 1 to 7 in cycles 1 through 6. Pembrolizumab was administered in a 30-min IV infusion every 3 weeks on day 1 of each 21-day cycle. Epacadostat tablets were administered orally twice daily.	Not available	2017–2020	[166]
Atezolizumab and guadecitabine	II (NCT03179943)	Suspended	Recurrent/advanced urothelial carcinoma (stage IV) patients who previously progressed on checkpoint inhibitor therapy with anti- PD-1 or PD-L1 therapy (*n* = 53)	Atezolizumab is administered intravenously on day 1 and day 22 of a 6-week cycle for a period of 8 cycles. Guadecitabine is administered subcutaneously on days 1 through 5 of the 6-week cycle for a period of 4 cycles.	Not available	2017–estimated end 2022	[167]

Abbreviations: 5-aza—5-azacytidine; DAC—decitabine.

## Supplementary Materials

The following are available online at https://www.mdpi.com/2073-4409/9/8/1850/s1: Figure S1. Chemical structure of nucleoside and non-nucleosides DNMT inhibitors.

## References

[1] Bray F., Ferlay J., Soerjomataram I., Siegel R.L., Torre L.A., Jemal A. (2018). Global cancer statistics 2018: GLOBOCAN estimates of incidence and mortality worldwide for 36 cancers in 185 countries. CA Cancer J. Clin..

[2] Kamat A.M., Hahn N.M., Efstathiou J.A., Lerner S.P., Malmström P.U., Choi W., Guo C.C., Lotan Y., Kassouf W. (2016). Bladder cancer. Lancet.

[3] Bellmunt J., Orsola A., Leow J.J., Wiegel T., De Santis M., Horwich A. (2014). Bladder cancer: ESMO Practice Guidelines for diagnosis, treatment and follow-up. Ann. Oncol..

[4] Amin M.B., Edge S., Greene F., Byrd D.R., Brookland R.K., Washington M.K., Gershenwald J.E., Compton C.C., Hess K.R., Sullivan D.C. (2017). AJCC Cancer Staging Manual.

[5] Redelman-Sidi G., Glickman M.S., Bochner B.H. (2014). The mechanism of action of BCG therapy for bladder cancer—A current perspective. Nat. Rev. Urol..

[6] Cancer Stat Facts: Bladder Cancer. https://seer.cancer.gov/statfacts/html/urinb.html.

[7] Mariotto A.B., Yabroff K.R., Shao Y., Feuer E.J., Brown M.L. (2011). Projections of the cost of cancer care in the United States: 2010–2020. J. Natl. Cancer Inst..

[8] Lobo J., Jerónimo C., Henrique R. (2020). Targeting the immune system and epigenetic landscape of urological tumors. Int. J. Mol. Sci..

[9] Hussain S.A., Birtle A., Crabb S., Huddart R., Small D., Summerhayes M., Jones R., Protheroe A. (2018). From clinical trials to real-life clinical practice: The role of immunotherapy with PD-1/PD-L1 inhibitors in advanced urothelial carcinoma. Eur. Urol. Oncol..

[10] Tan T.Z., Rouanne M., Tan K.T., Huang R.Y., Thiery J.P. (2019). Molecular subtypes of urothelial bladder cancer: Results from a meta-cohort analysis of 2411 tumors. Eur. Urol..

[11] Choi W., Ochoa A., McConkey D.J., Aine M., Höglund M., Kim W.Y., Real F.X., Kiltie A.E., Milsom I., Dyrskjøt L. (2017). Genetic alterations in the molecular subtypes of bladder cancer: Illustration in the cancer genome atlas dataset. Eur. Urol..

[12] Robertson A.G., Kim J., Al-Ahmadie H., Bellmunt J., Guo G., Cherniack A.D., Hinoue T., Laird P.W., Hoadley K.A., Akbani R. (2017). Comprehensive molecular characterization of muscle-invasive bladder cancer. Cell.

[13] Kelly A.D., Issa J.J. (2017). The promise of epigenetic therapy: Reprogramming the cancer epigenome. Curr. Opin. Genet. Dev..

[14] Baylin S.B., Jones P.A. (2011). A decade of exploring the cancer epigenome—Biological and translational implications. Nat. Rev. Cancer.

[15] Bennett R.L., Licht J.D. (2018). Targeting epigenetics in cancer. Annu. Rev. Pharmacol. Toxicol..

[16] Voyias P., Patel A., Arasaradnam R. (2016). Epigenetic biomarkers of disease. Medical Epigenetics.

[17] Inbar-Feigenberg M., Choufani S., Butcher D.T., Roifman M., Weksberg R. (2013). Basic concepts of epigenetics. Fertil. Steril..

[18] Porten S.P. (2018). Epigenetic alterations in bladder cancer. Curr. Urol. Rep..

[19] Duex J.E., Swain K.E., Dancik G.M., Paucek R.D., Owens C., Churchill M.E.A., Theodorescu D. (2018). Functional impact of chromatin remodeling gene mutations and predictive signature for therapeutic response in bladder cancer. Mol. Cancer Res..

[20] Cancer Genome Atlas Research Network (2014). Comprehensive molecular characterization of urothelial bladder carcinoma. Nature.

[21] Hurst C.D., Alder O., Platt F.M., Droop A., Stead L.F., Burns J.E., Burghel G.J., Jain S., Klimczak L.J., Lindsay H. (2017). Genomic subtypes of non-invasive bladder cancer with distinct metabolic profile and female gender bias in KDM6A mutation frequency. Cancer Cell.

[22] Ler L.D., Ghosh S., Chai X., Thike A.A., Heng H.L., Siew E.Y., Dey S., Koh L.K., Lim J.Q., Lim W.K. (2017). Loss of tumor suppressor KDM6A amplifies PRC2-regulated transcriptional repression in bladder cancer and can be targeted through inhibition of EZH2. Sci. Transl. Med..

[23] Ding B., Yan L., Zhang Y., Wang Z., Zhang Y., Xia D., Ye Z., Xu H. (2019). Analysis of the role of mutations in the KMT2D histone lysine methyltransferase in bladder cancer. FEBS Open Bio..

[24] Marques-Magalhães Â., Graça I., Henrique R., Jerónimo C. (2018). Targeting DNA Methyltranferases in Urological Tumors. Front. Pharmacol..

[25] Wolff E.M., Chihara Y., Pan F., Weisenberger D.J., Siegmund K.D., Sugano K., Kawashima K., Laird P.W., Jones P.A., Liang G. (2010). Unique DNA methylation patterns distinguish noninvasive and invasive urothelial cancers and establish an epigenetic field defect in premalignant tissue. Cancer Res..

[26] Wu H., Caffo B., Jaffee H.A., Irizarry R.A., Feinberg A.P. (2010). Redefining CpG islands using hidden Markov models. Biostatistics.

[27] Ferreira H.J., Esteller M. (2018). CpG Islands in Cancer: Heads, Tails, and Sides. Methods Mol. Biol..

[28] Rodríguez-Paredes M., Esteller M. (2011). Cancer epigenetics reaches mainstream oncology. Nat. Med..

[29] Kanwal R., Gupta S. (2012). Epigenetic modifications in cancer. Clin. Genet..

[30] Pasculli B., Barbano R., Parrella P. (2018). Epigenetics of breast cancer: Biology and clinical implication in the era of precision medicine. Semin. Cancer Biol..

[31] Lao V.V., Grady W.M. (2011). Epigenetics and colorectal cancer. Nat. Rev. Gastroenterol. Hepatol..

[32] Maunakea A.K., Chepelev I., Cui K., Zhao K. (2013). Intragenic DNA methylation modulates alternative splicing by recruiting MeCP2 to promote exon recognition. Cell Res..

[33] Maunakea A.K., Nagarajan R.P., Bilenky M., Ballinger T.J., D’Souza C., Fouse S.D., Johnson B.E., Hong C., Nielsen C., Zhao Y. (2010). Conserved role of intragenic DNA methylation in regulating alternative promoters. Nature.

[34] Bae M.G., Kim J.Y., Choi J.K. (2016). Frequent hypermethylation of orphan CpG islands with enhancer activity in cancer. BMC Med. Genom..

[35] Baubec T., Colombo D.F., Wirbelauer C., Schmidt J., Burger L., Krebs A.R., Akalin A., Schübeler D. (2015). Genomic profiling of DNA methyltransferases reveals a role for DNMT3B in genic methylation. Nature.

[36] Timinskas A., Butkus V., Janulaitis A. (1995). Sequence motifs characteristic for DNA [cytosine-N4] and DNA [adenine-N6] methyltransferases. Classification of all DNA methyltransferases. Gene.

[37] Lyko F. (2018). The DNA methyltransferase family: A versatile toolkit for epigenetic regulation. Nat. Rev. Genet..

[38] Gruenbaum Y., Cedar H., Razin A. (1982). Substrate and sequence specificity of a eukaryotic DNA methylase. Nature.

[39] Bestor T.H., Ingram V.M. (1983). Two DNA methyltransferases from murine erythroleukemia cells: Purification, sequence specificity, and mode of interaction with DNA. Proc. Natl. Acad. Sci. USA.

[40] Tahiliani M., Koh K.P., Shen Y., Pastor W.A., Bandukwala H., Brudno Y., Agarwal S., Iyer L.M., Liu D.R., Aravind L. (2009). Conversion of 5-methylcytosine to 5-hydroxymethylcytosine in mammalian DNA by MLL partner TET1. Science.

[41] Kohli R.M., Zhang Y. (2013). TET enzymes, TDG and the dynamics of DNA demethylation. Nature.

[42] Cedar H., Bergman Y. (2012). Programming of DNA methylation patterns. Annu. Rev. Biochem..

[43] Bhutani N., Burns D.M., Blau H.M. (2011). DNA demethylation dynamics. Cell.

[44] Baylin S.B., Jones P.A. (2016). Epigenetic determinants of cancer. Cold Spring Harb. Perspect. Biol..

[45] Hansen K.D., Timp W., Bravo H.C., Sabunciyan S., Langmead B., McDonald O.G., Wen B., Wu H., Liu Y., Diep D. (2011). Increased methylation variation in epigenetic domains across cancer types. Nat. Genet..

[46] Ross J.P., Rand K.N., Molloy P.L. (2010). Hypomethylation of repeated DNA sequences in cancer. Epigenomics.

[47] Rodriguez J., Frigola J., Vendrell E., Risques R.A., Fraga M.F., Morales C., Moreno V., Esteller M., Capellà G., Ribas M. (2006). Chromosomal instability correlates with genome-wide DNA demethylation in human primary colorectal cancers. Cancer Res..

[48] Saif I., Kasmi Y., Allali K., Ennaji M.M. (2018). Prediction of DNA methylation in the promoter of gene suppressor tumor. Gene.

[49] Kandimalla R., van Tilborg A.A., Zwarthoff E.C. (2013). DNA methylation-based biomarkers in bladder cancer. Nat. Rev. Urol..

[50] Larsen L.K., Lind G.E., Guldberg P., Dahl C. (2019). DNA-methylation-based detection of urological cancer in urine: Overview of biomarkers and considerations on biomarker design, source of DNA, and detection technologies. Int. J. Mol. Sci..

[51] Padrão N.A., Monteiro-Reis S., Torres-Ferreira J., Antunes L., Leça L., Montezuma D., Ramalho-Carvalho J., Dias P.C., Monteiro P., Oliveira J. (2017). MicroRNA promoter methylation: A new tool for accurate detection of urothelial carcinoma. Br. J. Cancer.

[52] Wu Y., Jiang G., Zhang N., Liu S., Lin X., Perschon C., Zheng S.L., Ding Q., Wang X., Na R. (2020). HOXA9, PCDH17, POU4F2, and ONECUT2 as a urinary biomarker combination for the detection of bladder cancer in Chinese patients with hematuria. Eur. Urol. Focus.

[53] Costa V.L., Henrique R., Danielsen S.A., Duarte-Pereira S., Eknaes M., Skotheim R.I., Rodrigues A., Magalhães J.S., Oliveira J., Lothe R.A. (2010). Three epigenetic biomarkers, GDF15, TMEFF2, and VIM, accurately predict bladder cancer from DNA-based analyses of urine samples. Clin. Cancer Res..

[54] Feber A., Dhami P., Dong L., de Winter P., Tan W.S., Martínez-Fernández M., Paul D.S., Hynes-Allen A., Rezaee S., Gurung P. (2017). UroMark-a urinary biomarker assay for the detection of bladder cancer. Clin. Epigenet..

[55] García-Baquero R., Puerta P., Beltran M., Alvarez-Mújica M., Alvarez-Ossorio J.L., Sánchez-Carbayo M. (2014). Methylation of tumor suppressor genes in a novel panel predicts clinical outcome in paraffin-embedded bladder tumors. Tumour Biol.

[56] Wang X., Chen E., Yang X., Wang Y., Quan Z., Wu X., Luo C. (2016). 5-azacytidine inhibits the proliferation of bladder cancer cells via reversal of the aberrant hypermethylation of the hepaCAM gene. Oncol. Rep..

[57] Erdmann A., Halby L., Fahy J., Arimondo P.B. (2015). Targeting DNA methylation with small molecules: What’s next?. J. Med. Chem..

[58] Bohl S.R., Bullinger L., Rücker F.G. (2018). Epigenetic therapy: Azacytidine and decitabine in acute myeloid leukemia. Expert Rev. Hematol..

[59] Stresemann C., Lyko F. (2008). Modes of action of the DNA methyltransferase inhibitors azacytidine and decitabine. Int. J. Cancer.

[60] Egger G., Liang G., Aparicio A., Jones P.A. (2004). Epigenetics in human disease and prospects for epigenetic therapy. Nature.

[61] Jones P.A., Taylor S.M. (1980). Cellular differentiation, cytidine analogues and DNA methylation. Cell.

[62] Levine A.J. (2017). The p53 protein plays a central role in the mechanism of action of epigentic drugs that alter the methylation of cytosine residues in DNA. Oncotarget.

[63] Nguyen A.N., Hollenbach P.W., Richard N., Luna-Moran A., Brady H., Heise C., MacBeth K.J. (2010). Azacitidine and decitabine have different mechanisms of action in non-small cell lung cancer cell lines. Lung Cancer (Auckl.).

[64] Datta J., Ghoshal K., Motiwala T., Jacob S.T. (2012). Novel insights into the molecular mechanism of action of DNA Hypomethylating agents: Role of protein kinase C δ in decitabine-induced degradation of DNA methyltransferase 1. Genes Cancer.

[65] Santi D.V., Norment A., Garrett C.E. (1984). Covalent bond formation between a DNA-cytosine methyltransferase and DNA containing 5-azacytosine. Proc. Natl. Acad. Sci. USA.

[66] Chen L., MacMillan A.M., Chang W., Ezaz-Nikpay K., Lane W.S., Verdine G.L. (1991). Direct identification of the active-site nucleophile in a DNA (cytosine-5)-methyltransferase. Biochemistry.

[67] Mortusewicz O., Schermelleh L., Walter J., Cardoso M.C., Leonhardt H. (2005). Recruitment of DNA methyltransferase I to DNA repair sites. Proc. Natl. Acad. Sci. USA.

[68] Welch J.S., Petti A.A., Miller C.A., Fronick C.C., O’Laughlin M., Fulton R.S., Wilson R.K., Baty J.D., Duncavage E.J., Tandon B. (2016). TP53 and decitabine in acute myeloid leukemia and myelodysplastic syndromes. N. Engl. J. Med..

[69] Gros C., Fahy J., Halby L., Dufau I., Erdmann A., Gregoire J.M., Ausseil F., Vispé S., Arimondo P.B. (2012). DNA methylation inhibitors in cancer: Recent and future approaches. Biochimie.

[70] Ganesan A., Arimondo P.B., Rots M.G., Jeronimo C., Berdasco M. (2019). The timeline of epigenetic drug discovery: From reality to dreams. Clin. Epigenet..

[71] Yoo C.B., Cheng J.C., Jones P.A. (2004). Zebularine: A new drug for epigenetic therapy. Biochem. Soc. Trans..

[72] Takemura Y., Satoh M., Hatanaka K., Kubota S. (2018). Zebularine exerts its antiproliferative activity through S phase delay and cell death in human malignant mesothelioma cells. Biosci. Biotechnol. Biochem..

[73] Cheng J.C., Yoo C.B., Weisenberger D.J., Chuang J., Wozniak C., Liang G., Marquez V.E., Greer S., Orntoft T.F., Thykjaer T. (2004). Preferential response of cancer cells to zebularine. Cancer Cell.

[74] Orta M.L., Pastor N., Burgos-Morón E., Domínguez I., Calderón-Montaño J.M., Huertas Castaño C., López-Lázaro M., Helleday T., Mateos S. (2017). Zebularine induces replication-dependent double-strand breaks which are preferentially repaired by homologous recombination. DNA Repair (Amst.).

[75] Liu H., Xue Z.T., Sjögren H.O., Salford L.G., Widegren B. (2007). Low dose Zebularine treatment enhances immunogenicity of tumor cells. Cancer Lett..

[76] Champion C., Guianvarc’h D., Sénamaud-Beaufort C., Jurkowska R.Z., Jeltsch A., Ponger L., Arimondo P.B., Guieysse-Peugeot A.L. (2010). Mechanistic insights on the inhibition of c5 DNA methyltransferases by zebularine. PLoS ONE.

[77] Gowher H., Jeltsch A. (2004). Mechanism of inhibition of DNA methyltransferases by cytidine analogues in cancer therapy. Cancer Biol. Ther..

[78] Beumer J.H., Eiseman J.L., Parise R.A., Joseph E., Holleran J.L., Covey J.M., Egorin M.J. (2006). Pharmacokinetics, metabolism, and oral bioavailability of the DNA methyltransferase inhibitor 5-fluoro-2′-deoxycytidine in mice. Clin. Cancer Res..

[79] Neil G.L., Moxley T.E., Kuentzel S.L., Manak R.C., Hanka L.J. (1975). Enhancement by tetrahydrouridine (NSC-112907) of the oral activity of 5-azacytidine (NSC-102816) in L1210 leukemic mice. Cancer Chemother. Rep..

[80] Beumer J.H., Parise R.A., Newman E.M., Doroshow J.H., Synold T.W., Lenz H.J., Egorin M.J. (2008). Concentrations of the DNA methyltransferase inhibitor 5-fluoro-2′-deoxycytidine (FdCyd) and its cytotoxic metabolites in plasma of patients treated with FdCyd and tetrahydrouridine (THU). Cancer Chemother. Pharmacol..

[81] Newman E.M., Morgan R.J., Kummar S., Beumer J.H., Blanchard M.S., Ruel C., El-Khoueiry A.B., Carroll M.I., Hou J.M., Li C. (2015). A phase I, pharmacokinetic, and pharmacodynamic evaluation of the DNA methyltransferase inhibitor 5-fluoro-2′-deoxycytidine, administered with tetrahydrouridine. Cancer Chemother. Pharmacol..

[82] Kantarjian H.M., Roboz G.J., Kropf P.L., Yee K.W.L., O’Connell C.L., Tibes R., Walsh K.J., Podoltsev N.A., Griffiths E.A., Jabbour E. (2017). Guadecitabine (SGI-110) in treatment-naive patients with acute myeloid leukaemia: Phase 2 results from a multicentre, randomised, phase 1/2 trial. Lancet Oncol..

[83] Lee V., Wang J., Zahurak M., Gootjes E., Verheul H.M., Parkinson R., Kerner Z., Sharma A., Rosner G., De Jesus-Acosta A. (2018). A phase I trial of a guadecitabine (SGI-110) and irinotecan in metastatic colorectal cancer patients previously exposed to irinotecan. Clin. Cancer Res..

[84] Garcia-Manero G., Roboz G., Walsh K., Kantarjian H., Ritchie E., Kropf P., O’Connell C., Tibes R., Lunin S., Rosenblat T. (2019). Guadecitabine (SGI-110) in patients with intermediate or high-risk myelodysplastic syndromes: Phase 2 results from a multicentre, open-label, randomised, phase 1/2 trial. Lancet Haematol..

[85] Laille E., Savona M.R., Scott B.L., Boyd T.E., Dong Q., Skikne B. (2014). Pharmacokinetics of different formulations of oral azacitidine (CC-486) and the effect of food and modified gastric pH on pharmacokinetics in subjects with hematologic malignancies. J. Clin. Pharmacol..

[86] Levy B.P., Giaccone G., Besse B., Felip E., Garassino M.C., Domine Gomez M., Garrido P., Piperdi B., Ponce-Aix S., Menezes D. (2019). Randomised phase 2 study of pembrolizumab plus CC-486 versus pembrolizumab plus placebo in patients with previously treated advanced non-small cell lung cancer. Eur. J. Cancer.

[87] Von Hoff D.D., Rasco D.W., Heath E.I., Munster P.N., Schellens J.H.M., Isambert N., Le Tourneau C., O’Neil B., Mathijssen R.H.J., Lopez-Martin J.A. (2018). Phase I study of CC-486 alone and in combination with carboplatin or nab-paclitaxel in patients with relapsed or refractory solid tumors. Clin. Cancer Res..

[88] Garcia-Manero G., Gore S.D., Kambhampati S., Scott B., Tefferi A., Cogle C.R., Edenfield W.J., Hetzer J., Kumar K., Laille E. (2016). Efficacy and safety of extended dosing schedules of CC-486 (oral azacitidine) in patients with lower-risk myelodysplastic syndromes. Leukemia.

[89] Peters G.J., Smid K., Vecchi L., Kathmann I., Sarkisjan D., Honeywell R.J., Losekoot N., Ohne O., Orbach A., Blaugrund E. (2013). Metabolism, mechanism of action and sensitivity profile of fluorocyclopentenylcytosine (RX-3117; TV-1360). Investig. New Drugs.

[90] Balboni B., El Hassouni B., Honeywell R.J., Sarkisjan D., Giovannetti E., Poore J., Heaton C., Peterson C., Benaim E., Lee Y.B. (2019). RX-3117 (fluorocyclopentenyl cytosine): A novel specific antimetabolite for selective cancer treatment. Expert Opin. Investig. Drugs.

[91] Yang M.Y., Lee Y.B., Ahn C.H., Kaye J., Fine T., Kashi R., Ohne O., Smid K., Peters G.J., Kim D.J. (2014). A novel cytidine analog, RX-3117, shows potent efficacy in xenograft models, even in tumors that are resistant to gemcitabine. Anticancer Res..

[92] Castillo-Aguilera O., Depreux P., Halby L., Arimondo P.B., Goossens L. (2017). DNA Methylation Targeting: The DNMT/HMT Crosstalk Challenge. Biomolecules.

[93] Pushpakom S., Iorio F., Eyers P.A., Escott K.J., Hopper S., Wells A., Doig A., Guilliams T., Latimer J., McNamee C. (2019). Drug repurposing: Progress, challenges and recommendations. Nat. Rev. Drug Discov..

[94] Villar-Garea A., Fraga M.F., Espada J., Esteller M. (2003). Procaine is a DNA-demethylating agent with growth-inhibitory effects in human cancer cells. Cancer Res..

[95] Moreira-Silva F., Camilo V., Gaspar V., Mano J.F., Henrique R., Jerónimo C. (2020). Repurposing old drugs into new epigenetic inhibitors: Promising candidates for cancer treatment?. Pharmaceutics.

[96] Li Y.C., Wang Y., Li D.D., Zhang Y., Zhao T.C., Li C.F. (2018). Procaine is a specific DNA methylation inhibitor with anti-tumor effect for human gastric cancer. J. Cell Biochem..

[97] Kuck D., Caulfield T., Lyko F., Medina-Franco J.L. (2010). Nanaomycin A selectively inhibits DNMT3B and reactivates silenced tumor suppressor genes in human cancer cells. Mol. Cancer Ther..

[98] Caulfield T., Medina-Franco J.L. (2011). Molecular dynamics simulations of human DNA methyltransferase 3B with selective inhibitor nanaomycin A. J. Struct. Biol..

[99] Dueñas-Gonzalez A., Coronel J., Cetina L., González-Fierro A., Chavez-Blanco A., Taja-Chayeb L. (2014). Hydralazine-valproate: A repositioned drug combination for the epigenetic therapy of cancer. Expert Opin. Drug Metab. Toxicol..

[100] Segura-Pacheco B., Trejo-Becerril C., Perez-Cardenas E., Taja-Chayeb L., Mariscal I., Chavez A., Acuña C., Salazar A.M., Lizano M., Dueñas-Gonzalez A. (2003). Reactivation of tumor suppressor genes by the cardiovascular drugs hydralazine and procainamide and their potential use in cancer therapy. Clin. Cancer Res..

[101] Graça I., Sousa E.J., Costa-Pinheiro P., Vieira F.Q., Torres-Ferreira J., Martins M.G., Henrique R., Jerónimo C. (2014). Anti-neoplastic properties of hydralazine in prostate cancer. Oncotarget.

[102] Pérez-Cárdenas E., Taja-Chayeb L., Trejo-Becerril C., Chanona-Vilchis J., Chávez-Blanco A., Domínguez-Gómez G., Langley E., García-Carrancá A., Dueñas-González A. (2018). Antimetastatic effect of epigenetic drugs, hydralazine and valproic acid, in Ras-transformed NIH 3T3 cells. Onco Targets Ther..

[103] Candelaria M., Gallardo-Rincón D., Arce C., Cetina L., Aguilar-Ponce J.L., Arrieta O., González-Fierro A., Chávez-Blanco A., de la Cruz-Hernández E., Camargo M.F. (2007). A phase II study of epigenetic therapy with hydralazine and magnesium valproate to overcome chemotherapy resistance in refractory solid tumors. Ann. Oncol..

[104] Coronel J., Cetina L., Pacheco I., Trejo-Becerril C., González-Fierro A., de la Cruz-Hernandez E., Perez-Cardenas E., Taja-Chayeb L., Arias-Bofill D., Candelaria M. (2011). A double-blind, placebo-controlled, randomized phase III trial of chemotherapy plus epigenetic therapy with hydralazine valproate for advanced cervical cancer. Preliminary results. Med. Oncol..

[105] Amato R.J. (2007). Inhibition of DNA methylation by antisense oligonucleotide MG98 as cancer therapy. Clin. Genitourin. Cancer.

[106] Davis A.J., Gelmon K.A., Siu L.L., Moore M.J., Britten C.D., Mistry N., Klamut H., D’Aloisio S., MacLean M., Wainman N. (2003). Phase I and pharmacologic study of the human DNA methyltransferase antisense oligodeoxynucleotide MG98 given as a 21-day continuous infusion every 4 weeks. Investig. New Drugs.

[107] Klisovic R.B., Stock W., Cataland S., Klisovic M.I., Liu S., Blum W., Green M., Odenike O., Godley L., Burgt J.V. (2008). A phase I biological study of MG98, an oligodeoxynucleotide antisense to DNA methyltransferase 1, in patients with high-risk myelodysplasia and acute myeloid leukemia. Clin. Cancer Res..

[108] Plummer R., Vidal L., Griffin M., Lesley M., de Bono J., Coulthard S., Sludden J., Siu L.L., Chen E.X., Oza A.M. (2009). Phase I study of MG98, an oligonucleotide antisense inhibitor of human DNA methyltransferase 1, given as a 7-day infusion in patients with advanced solid tumors. Clin. Cancer Res..

[109] Amato R.J., Stephenson J., Hotte S., Nemunaitis J., Bélanger K., Reid G., Martell R.E. (2012). MG98, a second-generation DNMT1 inhibitor, in the treatment of advanced renal cell carcinoma. Cancer Investig..

[110] Rondelet G., Fleury L., Faux C., Masson V., Dubois J., Arimondo P.B., Willems L., Wouters J. (2017). Inhibition studies of DNA methyltransferases by maleimide derivatives of RG108 as non-nucleoside inhibitors. Future Med. Chem..

[111] Yang L., Hou J., Cui X.H., Suo L.N., Lv Y.W. (2017). RG108 induces the apoptosis of endometrial cancer Ishikawa cell lines by inhibiting the expression of DNMT3B and demethylation of HMLH1. Eur Rev. Med. Pharmacol. Sci..

[112] Graça I., Sousa E.J., Baptista T., Almeida M., Ramalho-Carvalho J., Palmeira C., Henrique R., Jerónimo C. (2014). Anti-tumoral effect of the non-nucleoside DNMT inhibitor RG108 in human prostate cancer cells. Curr. Pharm. Des..

[113] Yoo J., Choi S., Medina-Franco J.L. (2013). Molecular modeling studies of the novel inhibitors of DNA methyltransferases SGI-1027 and CBC12: Implications for the mechanism of inhibition of DNMTs. PLoS ONE.

[114] Datta J., Ghoshal K., Denny W.A., Gamage S.A., Brooke D.G., Phiasivongsa P., Redkar S., Jacob S.T. (2009). A new class of quinoline-based DNA hypomethylating agents reactivates tumor suppressor genes by blocking DNA methyltransferase 1 activity and inducing its degradation. Cancer Res..

[115] Zwergel C., Schnekenburger M., Sarno F., Battistelli C., Manara M.C., Stazi G., Mazzone R., Fioravanti R., Gros C., Ausseil F. (2019). Identification of a novel quinoline-based DNA demethylating compound highly potent in cancer cells. Clin. Epigenet..

[116] Fang M.Z., Wang Y., Ai N., Hou Z., Sun Y., Lu H., Welsh W., Yang C.S. (2003). Tea polyphenol (−)-epigallocatechin-3-gallate inhibits DNA methyltransferase and reactivates methylation-silenced genes in cancer cell lines. Cancer Res..

[117] Nandakumar V., Vaid M., Katiyar S.K. (2011). (−)-Epigallocatechin-3-gallate reactivates silenced tumor suppressor genes, Cip1/p21 and p16INK4a, by reducing DNA methylation and increasing histones acetylation in human skin cancer cells. Carcinogenesis.

[118] Sheng J., Shi W., Guo H., Long W., Wang Y., Qi J., Liu J., Xu Y. (2019). The inhibitory effect of (−)-epigallocatechin-3-gallate on breast cancer progression via reducing SCUBE2 methylation and DNMT activity. Molecules.

[119] Bilir B., Sharma N.V., Lee J., Hammarstrom B., Svindland A., Kucuk O., Moreno C.S. (2017). Effects of genistein supplementation on genome-wide DNA methylation and gene expression in patients with localized prostate cancer. Int. J. Oncol..

[120] Xie Q., Bai Q., Zou L.Y., Zhang Q.Y., Zhou Y., Chang H., Yi L., Zhu J.D., Mi M.T. (2014). Genistein inhibits DNA methylation and increases expression of tumor suppressor genes in human breast cancer cells. Genes Chromosomes Cancer.

[121] Zhu J., Ren J., Tang L. (2018). Genistein inhibits invasion and migration of colon cancer cells by recovering WIF1 expression. Mol. Med. Rep..

[122] Link A., Balaguer F., Shen Y., Lozano J.J., Leung H.C., Boland C.R., Goel A. (2013). Curcumin modulates DNA methylation in colorectal cancer cells. PLoS ONE.

[123] Tao J., Liu Q., Wu X., Xu X., Zhang Y., Wang Q., Luo C. (2013). Identification of hypermethylation in hepatocyte cell adhesion molecule gene promoter region in bladder carcinoma. Int. J. Med. Sci..

[124] Hahn N.M., Bonney P.L., Dhawan D., Jones D.R., Balch C., Guo Z., Hartman-Frey C., Fang F., Parker H.G., Kwon E.M. (2012). Subcutaneous 5-azacitidine treatment of naturally occurring canine urothelial carcinoma: A novel epigenetic approach to human urothelial carcinoma drug development. J. Urol..

[125] Ramakrishnan S., Hu Q., Krishnan N., Wang D., Smit E., Granger V., Rak M., Attwood K., Johnson C., Morrison C. (2017). Decitabine, a DNA-demethylating agent, promotes differentiation via NOTCH1 signaling and alters immune-related pathways in muscle-invasive bladder cancer. Cell Death Dis..

[126] Maraver A., Fernandez-Marcos P.J., Cash T.P., Mendez-Pertuz M., Dueñas M., Maietta P., Martinelli P., Muñoz-Martin M., Martínez-Fernández M., Cañamero M. (2015). NOTCH pathway inactivation promotes bladder cancer progression. J. Clin. Investig..

[127] Zhang H., Qi F., Cao Y., Zu X., Chen M., Li Z., Qi L. (2013). 5-Aza-2′-deoxycytidine enhances maspin expression and inhibits proliferation, migration, and invasion of the bladder cancer T24 cell line. Cancer Biother. Radiopharm..

[128] Devanand P., Kim S.I., Choi Y.W., Sheen S.S., Yim H., Ryu M.S., Kim S.J., Kim W.J., Lim I.K. (2014). Inhibition of bladder cancer invasion by Sp1-mediated BTG2 expression via inhibition of DNA methyltransferase 1. FEBS J..

[129] Christoph F., Kempkensteffen C., Weikert S., Köllermann J., Krause H., Miller K., Schostak M., Schrader M. (2006). Methylation of tumour suppressor genes APAF-1 and DAPK-1 and in vitro effects of demethylating agents in bladder and kidney cancer. Br. J. Cancer.

[130] Cheng J.C., Weisenberger D.J., Gonzales F.A., Liang G., Xu G.L., Hu Y.G., Marquez V.E., Jones P.A. (2004). Continuous zebularine treatment effectively sustains demethylation in human bladder cancer cells. Mol. Cell Biol..

[131] Urakami S., Shiina H., Enokida H., Kawakami T., Tokizane T., Ogishima T., Tanaka Y., Li L.C., Ribeiro-Filho L.A., Terashima M. (2006). Epigenetic inactivation of Wnt inhibitory factor-1 plays an important role in bladder cancer through aberrant canonical Wnt/beta-catenin signaling pathway. Clin. Cancer Res..

[132] Liang G., Gonzales F.A., Jones P.A., Orntoft T.F., Thykjaer T. (2002). Analysis of gene induction in human fibroblasts and bladder cancer cells exposed to the methylation inhibitor 5-aza-2′-deoxycytidine. Cancer Res..

[133] Velicescu M., Weisenberger D.J., Gonzales F.A., Tsai Y.C., Nguyen C.T., Jones P.A. (2002). Cell division is required for de novo methylation of CpG islands in bladder cancer cells. Cancer Res..

[134] Chuang J.J., Dai Y.C., Lin Y.L., Chen Y.Y., Lin W.H., Chan H.L., Liu Y.W. (2014). Downregulation of glutathione S-transferase M1 protein in N-butyl-N-(4-hydroxybutyl)nitrosamine-induced mouse bladder carcinogenesis. Toxicol. Appl. Pharmacol..

[135] Kawakami T., Shiina H., Igawa M., Deguchi M., Nakajima K., Ogishima T., Tokizane T., Urakami S., Enokida H., Miura K. (2004). Inactivation of the hMSH3 mismatch repair gene in bladder cancer. Biochem. Biophys. Res. Commun..

[136] Giri A.K., Aittokallio T. (2019). DNMT Inhibitors increase methylation in the cancer genome. Front. Pharmacol..

[137] Yoo C.B., Jeong S., Egger G., Liang G., Phiasivongsa P., Tang C., Redkar S., Jones P.A. (2007). Delivery of 5-aza-2′-deoxycytidine to cells using oligodeoxynucleotides. Cancer Res..

[138] Chuang J.C., Warner S.L., Vollmer D., Vankayalapati H., Redkar S., Bearss D.J., Qiu X., Yoo C.B., Jones P.A. (2010). S110, a 5-Aza-2′-deoxycytidine–containing dinucleotide, is an effective DNA methylation inhibitor in vivo and can reduce tumor growth. Mol. Cancer Ther..

[139] Chen T., Hevi S., Gay F., Tsujimoto N., He T., Zhang B., Ueda Y., Li E. (2007). Complete inactivation of DNMT1 leads to mitotic catastrophe in human cancer cells. Nat. Genet..

[140] Wu C.T., Wu C.F., Lu C.H., Lin C.C., Chen W.C., Lin P.Y., Chen M.F. (2011). Expression and function role of DNA methyltransferase 1 in human bladder cancer. Cancer.

[141] Santoro S.W., Joyce G.F. (1997). A general purpose RNA-cleaving DNA enzyme. Proc. Natl. Acad. Sci. USA.

[142] Wang X., Zhang L., Ding N., Yang X., Zhang J., He J., Li Z., Sun L.Q. (2015). Identification and characterization of DNAzymes targeting DNA methyltransferase I for suppressing bladder cancer proliferation. Biochem Biophys. Res. Commun..

[143] Liu X., Dai X., Wu B. (2009). Study of 5-Aza-CdR on transcription regulation of RASSF1A gene in the BIU87 cell line. Urol. Int..

[144] Mori K., Enokida H., Kagara I., Kawakami K., Chiyomaru T., Tatarano S., Kawahara K., Nishiyama K., Seki N., Nakagawa M. (2009). CpG hypermethylation of collagen type I alpha 2 contributes to proliferation and migration activity of human bladder cancer. Int. J. Oncol..

[145] Matsumoto M., Kawakami K., Enokida H., Toki K., Matsuda R., Chiyomaru T., Nishiyama K., Kawahara K., Seki N., Nakagawa M. (2010). CpG hypermethylation of human four-and-a-half LIM domains 1 contributes to migration and invasion activity of human bladder cancer. Int. J. Mol. Med..

[146] Yoon H.Y., Kim Y.J., Kim J.S., Kim Y.W., Kang H.W., Kim W.T., Yun S.J., Ryu K.H., Lee S.C., Kim W.J. (2016). RSPH9 methylation pattern as a prognostic indicator in patients with non-muscle invasive bladder cancer. Oncol. Rep..

[147] Karam J.A., Fan J., Stanfield J., Richer E., Benaim E.A., Frenkel E., Antich P., Sagalowsky A.I., Mason R.P., Hsieh J.T. (2007). The use of histone deacetylase inhibitor FK228 and DNA hypomethylation agent 5-azacytidine in human bladder cancer therapy. Int. J. Cancer.

[148] Dhawan D., Ramos-Vara J.A., Hahn N.M., Waddell J., Olbricht G.R., Zheng R., Stewart J.C., Knapp D.W. (2013). DNMT1: An emerging target in the treatment of invasive urinary bladder cancer. Urol. Oncol..

[149] Wang C., Hamacher A., Petzsch P., Köhrer K., Niegisch G., Hoffmann M.J., Schulz W.A., Kassack M.U. (2020). Combination of decitabine and entinostat synergistically inhibits urothelial bladder cancer cells via activation of FoxO1. Cancers (Basel).

[150] Ramachandran K., Gordian E., Singal R. (2011). 5-azacytidine reverses drug resistance in bladder cancer cells. Anticancer Res..

[151] Khandelwal M., Anand V., Appunni S., Seth A., Singh P., Mathur S., Sharma A. (2018). Decitabine augments cytotoxicity of cisplatin and doxorubicin to bladder cancer cells by activating hippo pathway through RASSF1A. Mol. Cell Biochem..

[152] Xylinas E., Hassler M.R., Zhuang D., Krzywinski M., Erdem Z., Robinson B.D., Elemento O., Clozel T., Shariat S.F. (2016). An epigenomic approach to improving response to neoadjuvant cisplatin chemotherapy in bladder cancer. Biomolecules.

[153] Papafotiou G., Paraskevopoulou V., Vasilaki E., Kanaki Z., Paschalidis N., Klinakis A. (2016). KRT14 marks a subpopulation of bladder basal cells with pivotal role in regeneration and tumorigenesis. Nat. Commun..

[154] Zhu F., Qian W., Zhang H., Liang Y., Wu M., Zhang Y., Zhang X., Gao Q., Li Y. (2017). SOX2 Is a marker for stem-like tumor cells in bladder cancer. Stem Cell Rep..

[155] Wu M., Sheng L., Cheng M., Zhang H., Jiang Y., Lin S., Liang Y., Zhu F., Liu Z., Zhang Y. (2019). Low doses of decitabine improve the chemotherapy efficacy against basal-like bladder cancer by targeting cancer stem cells. Oncogene.

[156] Varol N., Konac E., Onen I.H., Gurocak S., Alp E., Yilmaz A., Menevse S., Sozen S. (2014). The epigenetically regulated effects of Wnt antagonists on the expression of genes in the apoptosis pathway in human bladder cancer cell line (T24). DNA Cell Biol..

[157] San José-Enériz E., Agirre X., Rabal O., Vilas-Zornoza A., Sanchez-Arias J.A., Miranda E., Ugarte A., Roa S., Paiva B., Estella-Hermoso de Mendoza A. (2017). Discovery of first-in-class reversible dual small molecule inhibitors against G9a and DNMTs in hematological malignancies. Nat. Commun..

[158] Bárcena-Varela M., Caruso S., Llerena S., Álvarez-Sola G., Uriarte I., Latasa M.U., Urtasun R., Rebouissou S., Alvarez L., Jimenez M. (2019). Dual targeting of histone methyltransferase G9a and DNA-methyltransferase 1 for the treatment of experimental hepatocellular carcinoma. Hepatology.

[159] Segovia C., San José-Enériz E., Munera-Maravilla E., Martínez-Fernández M., Garate L., Miranda E., Vilas-Zornoza A., Lodewijk I., Rubio C., Segrelles C. (2019). Inhibition of a G9a/DNMT network triggers immune-mediated bladder cancer regression. Nat. Med..

[160] Shang D., Liu Y., Matsui Y., Ito N., Nishiyama H., Kamoto T., Ogawa O. (2008). Demethylating agent 5-aza-2′-deoxycytidine enhances susceptibility of bladder transitional cell carcinoma to Cisplatin. Urology.

[161] Bunch B., Krishnan N., Greenspan R.D., Ramakrishnan S., Attwood K., Yan L., Qi Q., Wang D., Morrison C., Omilian A. (2019). TAp73 expression and P1 promoter methylation, a potential marker for chemoresponsiveness to cisplatin therapy and survival in muscle-invasive bladder cancer (MIBC). Cell Cycle.

[162] Coyne G.O., Wang L., Zlott J., Juwara L., Covey J.M., Beumer J.H., Cristea M.C., Newman E.M., Koehler S., Nieva J.J. (2020). Intravenous 5-fluoro-2′-deoxycytidine administered with tetrahydrouridine increases the proportion of p16-expressing circulating tumor cells in patients with advanced solid tumors. Cancer Chemother. Pharmacol..

[163] Lin J., Gilbert J., Rudek M.A., Zwiebel J.A., Gore S., Jiemjit A., Zhao M., Baker S.D., Ambinder R.F., Herman J.G. (2009). A phase I dose-finding study of 5-azacytidine in combination with sodium phenylbutyrate in patients with refractory solid tumors. Clin. Cancer Res..

[164] A Phase 1 Study of CC-486 as a Single Agent and in Combination with Carboplatin or ABI-007 in Subjects with Relapsed or Refractory Solid Tumors. https://clinicaltrials.gov/ct2/show/results/NCT01478685.

[165] LoRusso P., Rasco D., Bendell J., Sachdev J., Ramanathan R., Weiss G., Munster P., Edenfield W.J., Liu K., Blackwood-Chirchir A. (2013). Abstract A120: A phase Ib study of CC-486 (oral azacitidine) as a priming agent for carboplatin or NAB-paclitaxel in subjects with relapsed and refractory solid tumors. Mol. Cancer Ther..

[166] Azacitidine Combined with Pembrolizumab and Epacadostat in Subjects with Advanced Solid Tumors (ECHO-206). https://clinicaltrials.gov/ct2/show/results/NCT02959437.

[167] Atezolizumab + Guadecitabine in Patients with Checkpoint Inhibitor Refractory or Resistant Urothelial Carcinoma. https://clinicaltrials.gov/ct2/show/results/NCT03179943.

[168] Decitabine in Treating Patients with Advanced Solid Tumors. https://clinicaltrials.gov/ct2/show/study/NCT00030615.

[169] A Multi-Histology Phase II Study of 5-Fluoro-2′-Deoxycytidine with Tetrahydrouridine (FdCyd + THU). https://clinicaltrials.gov/ct2/show/results/NCT00978250.

[170] Dose-Finding and Safety Study for Oral Single-Agent to Treat Advanced Malignancies. https://clinicaltrials.gov/ct2/show/results/NCT02030067.

[171] Bioequivalence & Food Effect Study in Patients with Solid Tumor or Hematologic Malignancies. https://clinicaltrials.gov/ct2/show/study/NCT02223052.

[172] Crabb S., Danson S.J., Catto J.W.F., McDowell C., Lowder J.N., Caddy J., Dunkley D., Rajaram J., Ellis D., Hill S. (2018). SPIRE—Combining SGI-110 with cisplatin and gemcitabine chemotherapy for solid malignancies including bladder cancer: Study protocol for a phase Ib/randomised IIa open label clinical trial. Trials.

[173] Genomic Based Assignment of Therapy in Advanced Urothelial Carcinoma. https://clinicaltrials.gov/ct2/show/study/NCT02788201.

