# Supplementary material for: DNA Methylation as a Therapeutic Target for Bladder Cancer
Source: Cells. 2020 Aug 7;9(8):1850. doi: 10.3390/cells9081850 (PMC7463638; doi:10.3390/cells9081850)
Supplement: Supplementary file 1 [file cells-09-01850-s001.pdf]

Nucleoside analogues

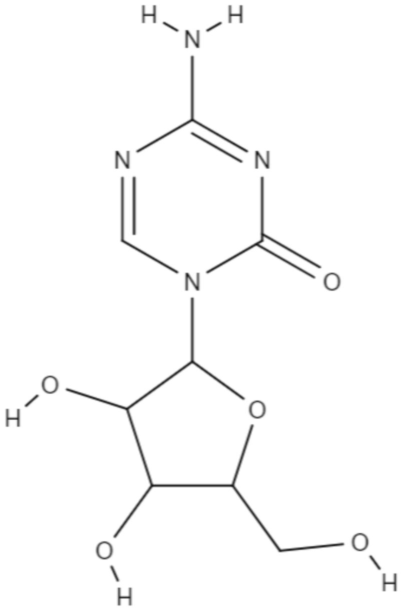

5-azacytidine (5-aza)

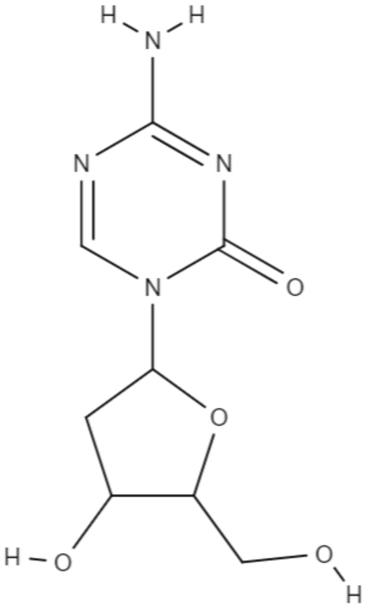

Decitabine (DAC)

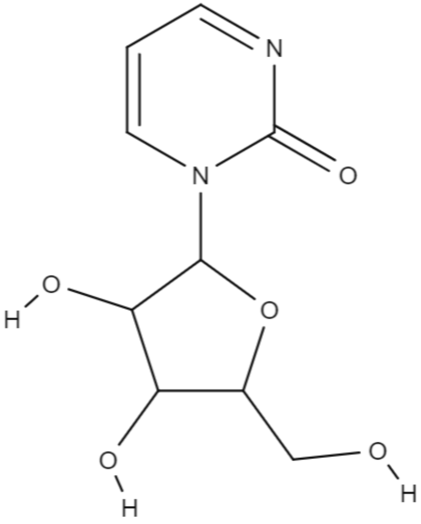

Zebularine

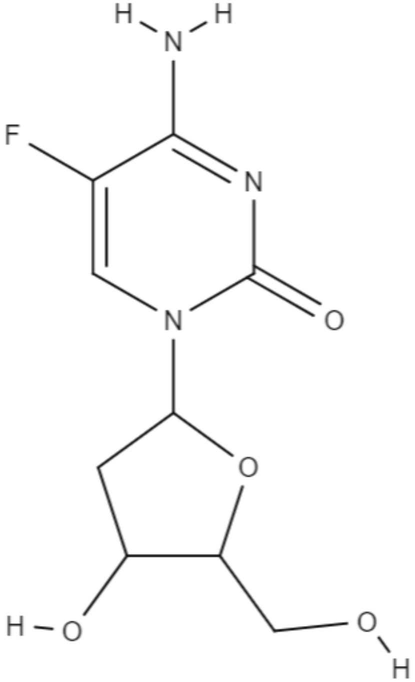

5’-fluoro-2’-deoxycytidine (FdCyd)

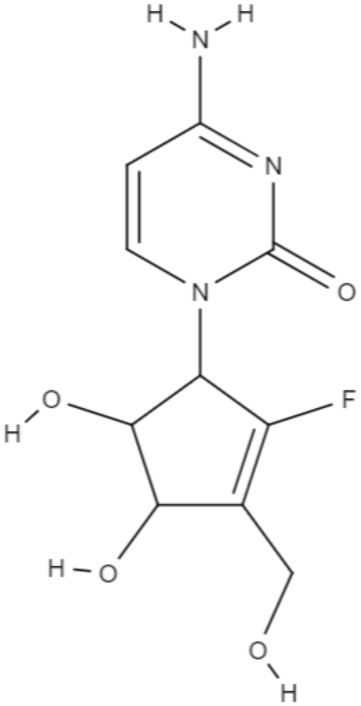

RX-3117

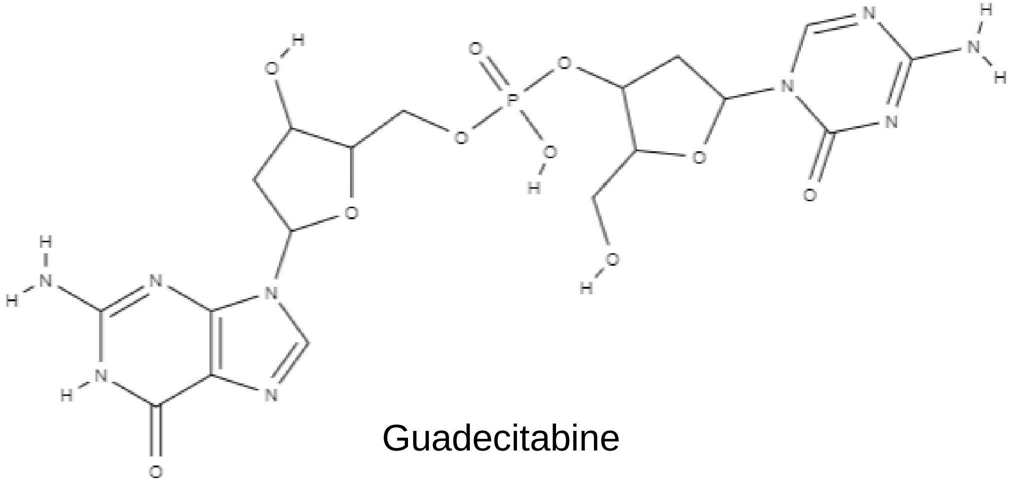

Guadecitabine

Non-nucleoside analogues

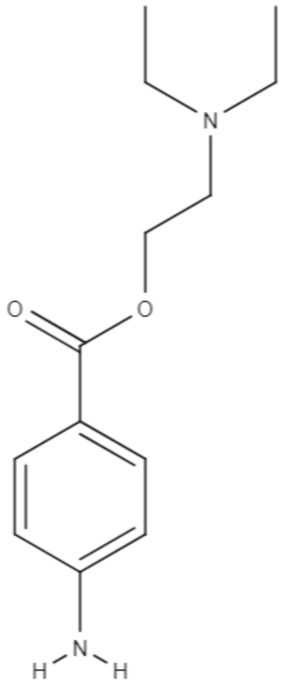

Procaine

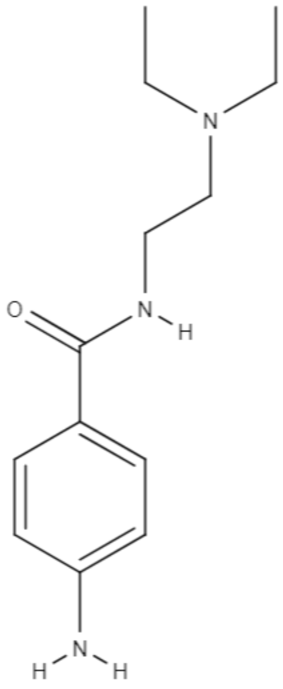

Procainamide

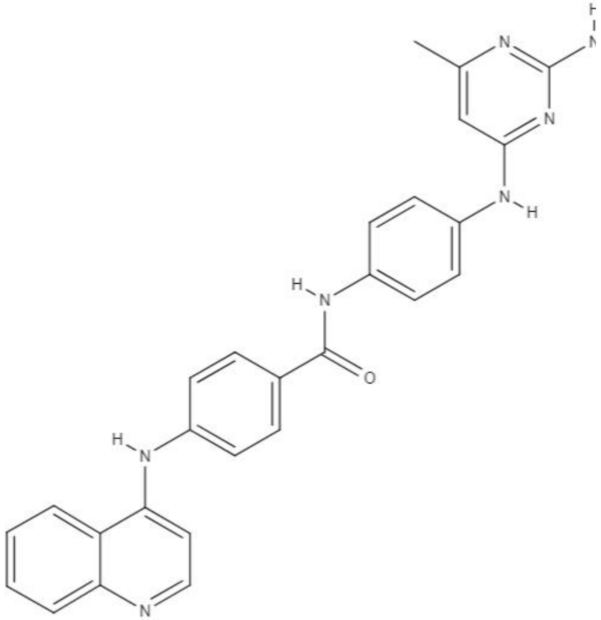

SGI-1027

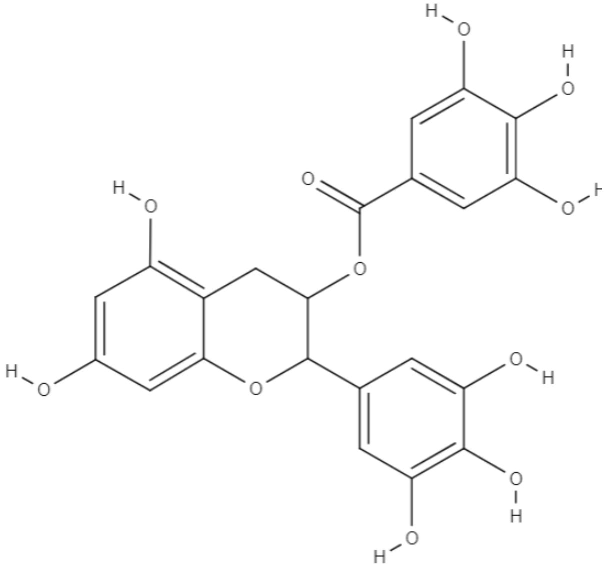

Epigallocatechin-3-gallate (EGCG)

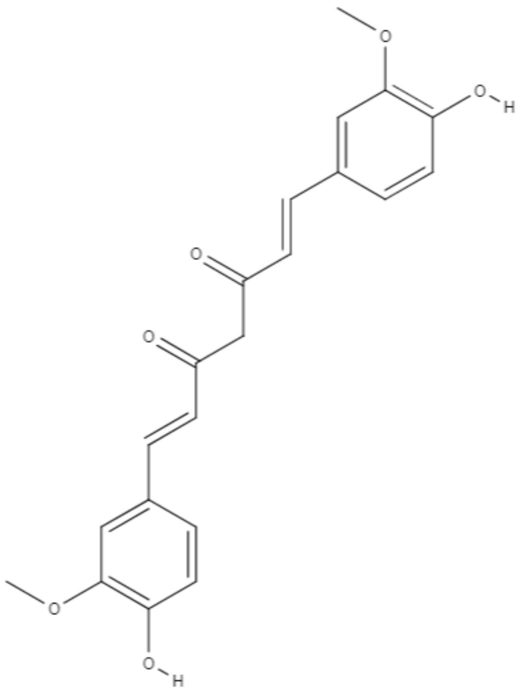

Curcumin

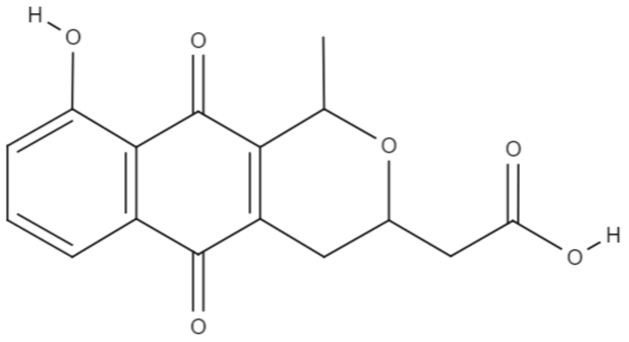

Nanaomycin A

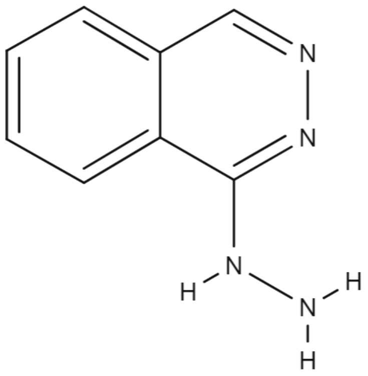

Hydralazine

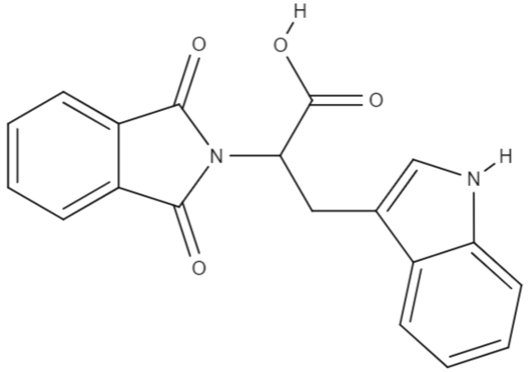

RG108

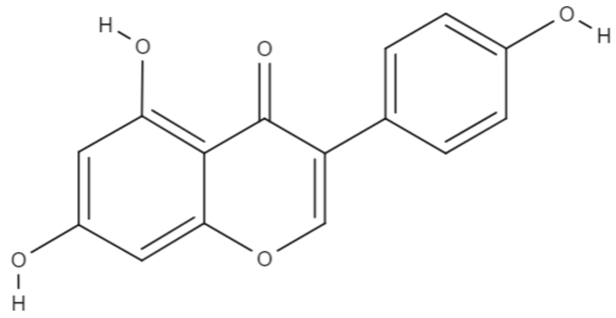

Genistein
